# Global Registration of Subway Tunnel Point Clouds Using an Augmented Extended Kalman Filter and Central-Axis Constraint

**DOI:** 10.1371/journal.pone.0126862

**Published:** 2015-05-12

**Authors:** Zhizhong Kang, Jinlei Chen, Baoqian Wang

**Affiliations:** 1 School of Land Science and Technology, China University of Geosciences, Beijing, China; 2 Beijing Siwei Spatial Data Technology Co., Ltd., Beijing, China; Fondazione Edmund Mach, Research and Innovation Centre, ITALY

## Abstract

Because tunnels generally have tubular shapes, the distribution of tie points between adjacent scans is usually limited to a narrow region, which makes the problem of registration error accumulation inevitable. In this paper, a global registration method is proposed based on an augmented extended Kalman filter and a central-axis constraint. The point cloud registration is regarded as a stochastic system, and the global registration is considered to be a process that recursively estimates the rigid transformation parameters between each pair of adjacent scans. Therefore, the augmented extended Kalman filter (AEKF) is used to accurately estimate the rigid transformation parameters by eliminating the error accumulation caused by the pair-wise registration. Moreover, because the scanning range of a terrestrial laser scanner can reach hundreds of meters, a single scan can cover a tunnel segment with a length of more than one hundred meters, which means that the central axis extracted from the scan can be employed to control the registration of multiple scans. Therefore, the central axis of the subway tunnel is first determined through the 2D projection of the tunnel point cloud and curve fitting using the RANSAC (RANdom SAmple Consensus) algorithm. Because the extraction of the central axis by quadratic curve fitting may suffer from noise in the tunnel points and from variations in the tunnel, we present a global extraction algorithm that is based on segment-wise quadratic curve fitting. We then derive the central-axis constraint as an additional observation model of AEKF to optimize the registration parameters between each pair of adjacent scans. The proposed approach is tested on terrestrial point clouds that were acquired in a subway tunnel. The results show that the proposed algorithm is capable of improving the accuracy of aligning multiple scans by 48%.

## Introduction

Because underground structures, such as tunnels, require routine inspections and maintenance for their optimal use, efficient and accurate tunnel inspections are mandatory. The construction of 3D models of tunnels is important for such applications. Applications of laser technology are rapidly expanding with decreased costs and increased accuracy. Therefore, 3D laser scanners make it possible to obtain point clouds with high accuracy and high spatial resolution for 3D tunnel model construction. However, one of the biggest problems that are encountered when processing these scans is terrestrial point cloud registration, in which rigid transformation parameters (RTPs) are used to align one dataset with another. Because tunnels generally have tubular shapes, the distribution of the tie points between adjacent scans is usually limited to a narrow region. Therefore, the problem of registration error accumulation becomes inevitable when multiple scans must be registered. Bergevin et al. [1] presented an algorithm that considers the network of views as a whole and minimizes the registration errors of all views simultaneously. Inspired by that work, Benjemaa and Schmitt [2] extended pair-wise registration based on a multi-z-buffer technique to a global registration. They applied rigid transformations to transform each moving surface immediately after its rigid transformation had been estimated. Similarly, Sharp et al. [3] proposed an analytical method to solve for global registration parameters that involves building a graph to describe the relationship between neighboring views. This approach then decomposes the graph into basis cycles so the nonlinear optimization problem can be solved over each basis cycle in a closed form. Hu et al. [4] built a topological graph to determine the best registration path for all range scans. Stoddart and Hilton [5] identified pair-wise correspondences between points in all views and then iteratively minimized the correspondence errors over all views using a descent algorithm. This basic technique was extended by Neugebauer [6] and Eggert et al. [7] using a multiresolution framework, surface normal processing, and boundary point processing. Williams and Bennamoun [8] suggested a further refinement by including individual covariance weights for each point. There is currently no consensus as to the best approach for solving the global registration problem. Kang et al. [9] proposed a global registration method that minimizes the self-closure errors across all scans through simultaneous least-squares adjustments. Additional sensors, such as Global Navigation Satellite System (GNSS), compasses, and tilt sensors, are often combined with TLS instruments to help solve or reduce the global registration problem.

In recent years, optimization algorithms that use a series of measurements observed over time have been introduced in point cloud registration. Ma and Ellis [10] proposed the unscented particle filter (UPF) algorithm [11] to register two point data sets in the presence of isotropic Gaussian noise. Moghari and Abolmaesumi [12] proposed a registration algorithm, the unscented Kalman filter (UKF) [13], which is based on the continuous assessment of point cloud registration parameters of two rigid bodies. In this paper, we regard the point cloud registration as a stochastic system and the global registration as a process that recursively estimates the rigid transformation parameters of each scan. The augmented extended Kalman filter (AEKF) is utilized to produce accurate estimates of rigid transformation parameters by eliminating the error accumulation that is caused by the pair-wise registration. Because the central axis extracted from a single scan predictably controls more than the tie points, it is employed to reduce the error accumulation for the registration of multiple scans. Therefore, the central-axis constraints are derived as the control condition of the AEKF, so the registration parameters between each pair of adjacent scans will be globally optimized.

We begin by proposing the global registration method based on the augmented extended Kalman filter in Section 2. Section 3 optimizes the AEKF by introducing the central-axis constraint as an additional observation model. Section 4 discusses the test results, and we offer conclusions and suggestions for further research in Section 5.

## Global Registration Using an Augmented Extended Kalman Filter

The Kalman filter, which is also known as linear quadratic estimation (LQE), is an algorithm that uses a series of measurements observed over time, which contain noise (random variations) and other inaccuracies, and produces estimates of unknown variables that tend to be more precise than those that are based on a single measurement.

In this paper, the point cloud registration is regarded as a stochastic system, and the global registration is the process that recursively estimates the rigid transformation parameters of each scans. Therefore, a Kalman filter is used to produce accurate estimates of the rigid transformation parameters by eliminating the error accumulation that is caused by the pair-wise registration.

Because the rigid transformation model is nonlinear, we utilize the extended Kalman filter (EKF) [14], which is the nonlinear version of the Kalman filter and is the de facto standard in the theory of nonlinear state estimation, navigation systems and GPS, to estimate the six rigid transformation parameters (three for the translation and three for the rotation). As a global registration process, the RTPs that are acquired by pair-wise registrations should be globally optimized. Therefore, the system state is augmented to contain the RTPs of all pair-wise registrations that have been completed, so the optimized RTPs in the global reference frame are estimated in terms of the RTPs of the new registration and its preceding registration. This paper presents a design for an augmented extended Kalman filter (AEKF) for the global registration of tunnel point clouds.

Suppose that N scans of 3D point cloud data are expressed as: {V_1_, V_2_,…, V_N_}. The i-th scan V_i_ is represented as {v_ij_ = (x_ij_, y_ij_, z_ij_) | j = 1,2,…,M_i_}, and M_i_ represents the point number of the i-th scan.

For a scale factor of 1, the rigid transformation between adjacent scans is parameterized as follows:

$$\left[\begin{array}{l} X\prime \\ Y\prime \\ Z\prime \end{array}\right]=R\left[\begin{array}{l} X\\ Y\\ Z \end{array}\right]\;+\;\left[\begin{array}{l} T_{X}\\ T_{Y}\\ T_{Z} \end{array}\right]$$ (1)

where (*X*, *Y*, *Z*) and (*X’*, *Y’*, *Z’*) are the coordinates of the corresponding points in the analyzed and fixed scans, respectively, *R* is the rotation matrix computed by three rotations around the coordinate axes *φ*,*ω*,*κ*, and (*T*_*X*_, *T*_*Y*_, *T*_*Z*_) is the translation vector.

Therefore, each transformation has six degrees of freedom (6DOF): *T*_*X*_,*T*_*Y*_,*T*_*Z*_,*φ*,*ω*,*κ*.

### State space and system models

When using the Kalman filter to estimate the RTPs, the system state model, observation model and extended model should be defined first.

#### System state space

The augmented system state comprises the RTPs of all of the completed pair-wise registrations. The system state at time tk is defined as X(k):

$$X(k)=\left[\begin{array}{l} \underset{\begin{array}{l} .\\ .\\ . \end{array}}{X_{1}(k)}\\ X_{n}(k) \end{array}\right]\;X_{i}(k)=\left[\begin{array}{l} T_{Xi}(k)\\ T_{Yi}(k)\\ T_{Zi}(k)\\ \varphi_{i}(k)\\ \omega_{i}(k)\\ \kappa_{i}(k) \end{array}\right]$$ (2)

where n represents the number of completed pair-wise registrations, and Xi(k) denotes the RTPs of the i-th pair-wise registration.

The system covariance matrix is a symmetric matrix that is expressed as

$$P\left(k\right)=\left[\begin{array}{l} p_{11}\left(k\right)\;\cdots \;p_{1n}\left(k\right)\\ \;\vdots \;\ddots \;\vdots \\ p_{11}\left(k\right)\;\cdots \;p_{nn}\left(k\right) \end{array}\right]$$ (3)

where p_ij_ (k) is a block matrix that represents the covariance matrix between X_i_(k) and X_j_(k).

#### System status model

Because the RTPs of each pair-wise registration are static, the system state transition equation becomes

X(k + 1)=f (X(k))=X(k) (4)

where f(.) is the state-transition model. The state-transition model is a unit matrix I, which can be ignored.

### System augmented model

During the global registration process, when a new pair-wise registration is considered at time tk, its RTPs are added to the system state vector. The RTPs in the global reference frame are estimated in terms of the RTPs of the new registration and its preceding registration using Eq (5).

$$X_{new}\left(k\right)=g\;\left(X_{r}\left(k-1\right),\;T_{M}\right)+\omega \left(k\right)$$ (5)

where *X*_*new*_(*k*) represents the augmented RTPs of the currently considered registration, *X*_*r*_(*k*−1) denotes the RTPs of the preceding registration, g(.) is a system-augmented function, the RTPs of the new pair-wise registration are *T*_*M*_ = [Δ*T*_*X*_ Δ*T*_*Y*_ Δ*T*_*Z*_ Δ*φ* Δ*ω* Δ*κ*], and ω(k) describes a variety of uncertainties in the pair-wise registration and modeling process, which is assumed to comply with the Gaussian distribution and is thus expressed as a white noise vector N (0, Q).

### Observation model

An observation model is established to optimize the RTPs of all of the pair-wise registrations by minimizing the differences between the corresponding 3D point pairs transformed into the common reference frame. Therefore, the observation value Z is the difference between a corresponding point pair, so the observation model is derived as

$$Z_{P}=h_{P}\left(X_{m}\left(k\right),\ldots ,\;X_{n}\left(k\right)\right)\;+\upsilon \left(k\right)=\left[\begin{array}{l} X_{i\;f}\\ Y_{i\;f}\\ Z_{i\;f} \end{array}\right]-\left[\begin{array}{l} X_{i\;a}\prime \\ Y_{i\;a}\prime \\ Z_{i\;a}\prime \end{array}\right]+\upsilon \left(k\right)$$ (6)

where (*X*_*if*_, *Y*_*if*_, *Z*_*if*_) and ($X_{i\;a}\prime$, $Y_{i\;a}\prime$,$Z_{i\;a}\prime$) are the coordinates of the corresponding point pair i in the fixed and analyzed scans, respectively, transformed into the common reference frame, *h*(.) is the observation model, and *υ*(*k*) denotes a variety of uncertainties in the scanning measurement and the transformation of coordinates, which is assumed to comply with the Gaussian distribution and is thus expressed as a white noise vector N (0, R).

The coordinates ($X_{i\;a}\prime$, $Y_{i\;a}\prime$,$Z_{i\;a}\prime$) are computed as follows:

$$\left[\begin{array}{l} X_{i\;a}\prime \\ Y_{i\;a}\prime \\ Z_{i\;a}\prime \end{array}\right]=R_{m}\cdots \;R_{n}\left[\begin{array}{l} X_{i\;a}\\ Y_{i\;a}\\ Z_{i\;a} \end{array}\right]+\left[\begin{array}{l} T_{m,\;X}\\ T_{m,\;Y}\\ T_{m,\;Z} \end{array}\right]+R_{m}\left[\begin{array}{l} T_{m\;+\;1,\;X}\\ T_{m\;+\;1,\;Y}\\ T_{m\;+\;1,\;Z} \end{array}\right]+\cdots +R_{m}\cdots R_{n\;-\;1}\left[\begin{array}{l} T_{n,\;X}\\ T_{n,\;Y}\\ T_{n,\;Z} \end{array}\right]$$ (7)

where (*X*_*ia*_, *Y*_*ia*_, *Z*_*ia*_) are the coordinates of the point in the analyzed scan, (*T*_*i*,*X*_, *T*_*i*,*Y*_, *T*_*i*,*Z*_) represent the translation from scan i+1 to i, and R_i_ is the rotation from i+1 to i.

Eq (6) is linearized as

$$Z_{P}=h_{P}{}^{0}\left(X_{m}\left(k\right),\;\ldots \;,\;X_{n}\left(k\right)\right)+\nabla h_{P}*\left[\begin{array}{l} \Delta X_{m}\left(k\right)\\ \;\vdots \\ \Delta X_{n}\left(k\right) \end{array}\right]+\upsilon \left(k\right)$$ (8)

where ∇*h*_*P*_ is the Jacobian matrix derived from Eqs (6) and (7).

$$\begin{array}{l} {\left[\begin{array}{cccccc} \Delta T_{X_{n}}(k) & \Delta T_{Y_{n}}(k) & \Delta T_{Z_{n}}(k) & \Delta \phi_{n}(k) & \Delta \omega_{n}(k) & \Delta \kappa_{n}(k) \end{array}\right]}^{T}\\ \Delta X_{m}(k)={\left[\begin{array}{cccccc} \Delta T_{X_{m}}(k) & \Delta T_{Y_{m}}(k) & \Delta T_{Z_{m}}(k) & \Delta \phi_{m}(k) & \Delta \omega_{m}(k) & \Delta \kappa_{m}(k) \end{array}\right]}^{T} \end{array}\}$$ (9)

### Status augmentation

When a new pair-wise registration is completed, *X*_*new*_(*k*) is added to the system state vector. The system state vector and the system covariance matrices are then augmented as follows:

$$\begin{array}{l} \;X^{-}\left(k\right)=\left[\begin{array}{l} X^{+}\left(k-1\right)\\ X_{new}\;\left(k\right) \end{array}\right]\\ \;P^{-}\left(k\right)=\left[\begin{array}{l} P^{+}\left(k\;-\;1\right){\left(\nabla g*P^{+}\left(k-1\right)\right)}^{T}\\ \nabla g*P^{+}\left(k-1\right)\nabla g*P^{+}\left(k-1\right)\;*\nabla g^{T}+Q \end{array}\right] \end{array}\}$$ (10)

where X_new_ denotes the augmented RTPs of the new pair-wise registration, X^+^ and P^+^ represent the a posteriori system state estimation, X^-^ and P^-^ represent the a priori system state estimation, ∇g is the Jacobian matrix of the system augmented model, and *Q* is the covariance matrix of the system noise.

### Observation updates

In terms of the observation model and the augmented system state vector, the new information ∨(*k*), the new information variance *S*(*k*) and the Kalman gain *W* are computed as follows:

$$\begin{array}{l} \vee \left(k\right)=0-\left(h\left(X_{i}^{-}\left(k\right),X_{j}^{-}\left(k\right)\right)+\upsilon \left(k\right)\right)\\ S\left(k\right)=\nabla h*P^{-}\left(k\right)*\nabla h^{T}+R\\ W=P^{-}\left(k\right)*\nabla h^{T}*S{\left(k\right)}^{-1} \end{array}\}$$ (11)

The state vector and covariance matrix of the system are updated as

$$\begin{array}{l} X^{+}\left(k\right)=X^{-\;}\left(k\right)+W*\vee \left(k\right)\\ P^{+}\left(k\right)=P^{-\;}\left(k\right)-W*S\left(k\right)*W^{T} \end{array}\}$$ (12)

where *X* ^+^ and *P* ^+^ represent the a posteriori system state estimation, and *X* ^-^ and *P* ^-^ represent the a priori system state estimation.

## AEKF with Central-Axis Constraint

**Fig 1:**
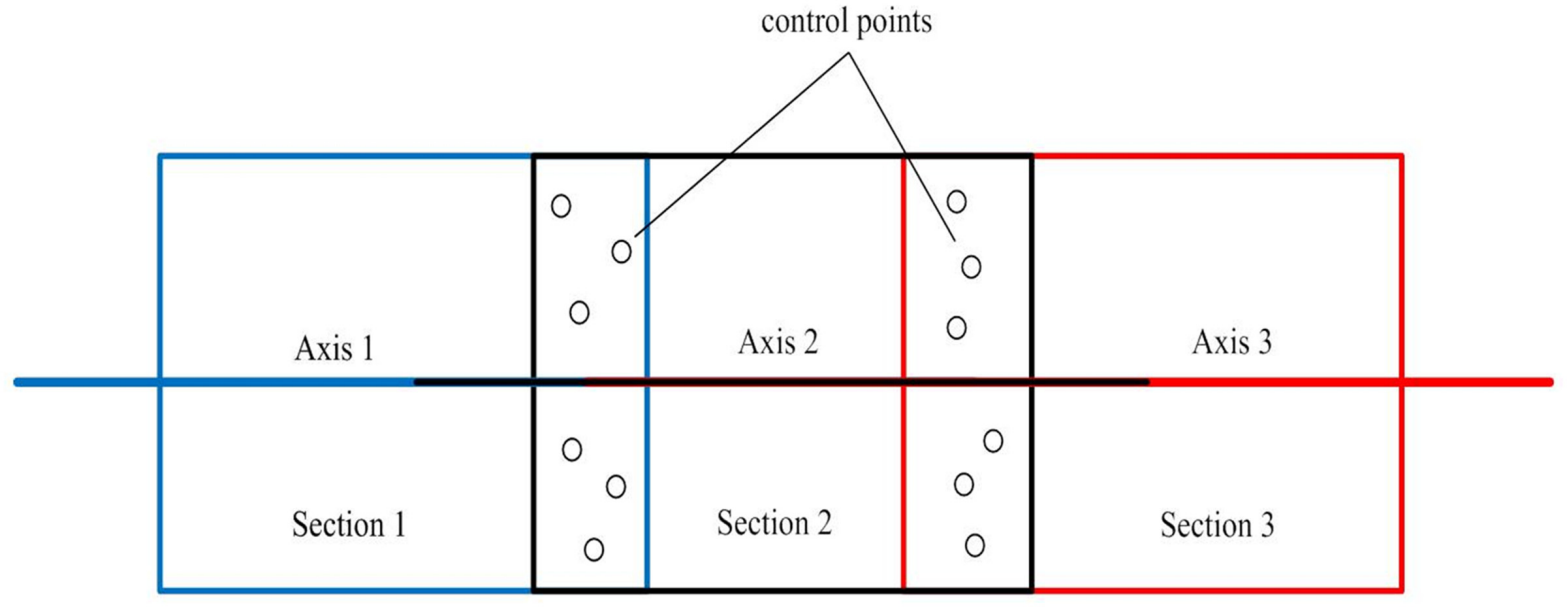
Overlaps of the central axes. The overlap between the fitted axes is much larger than that between the tie points.

Because tunnels generally have tubular shapes, the distribution of tie points between adjacent scans is usually limited to a narrow region. As a result, the registration of point clouds is prone to error accumulation. The central axis that is extracted from a single scan can be more than 100 meters long, while the shift between adjacent scans in a tunnel has to be short (e.g., 20 meters) because long shifts between adjacent scans will lead to large differences between the corresponding scanning angles of incidence to the same object point, which will affect the accuracy of the identification of tie points for the registration. Therefore, the overlap between the fitted axes is expected to be much larger than that between the tie points (Fig 1), from which a constraint can be derived to control the error accumulation.

First, we propose a global extraction algorithm that is based on segment-wise quadratic curve fitting to extract the central axis of the subway tunnel. The central-axis constraint is then derived as an additional observation model of the AEKF to optimize the registration parameters between each pair of adjacent scans.

### Determination of the central axis of a tunnel based on 2D projections

The central axis of a tunnel is continuously extracted using a 2D projection of the point cloud and curve fitting using the RANSAC algorithm, and the axis is optimized using a global extraction strategy that is based on segment-wise fitting.

#### Estimation of the boundary points

**Fig 2:**
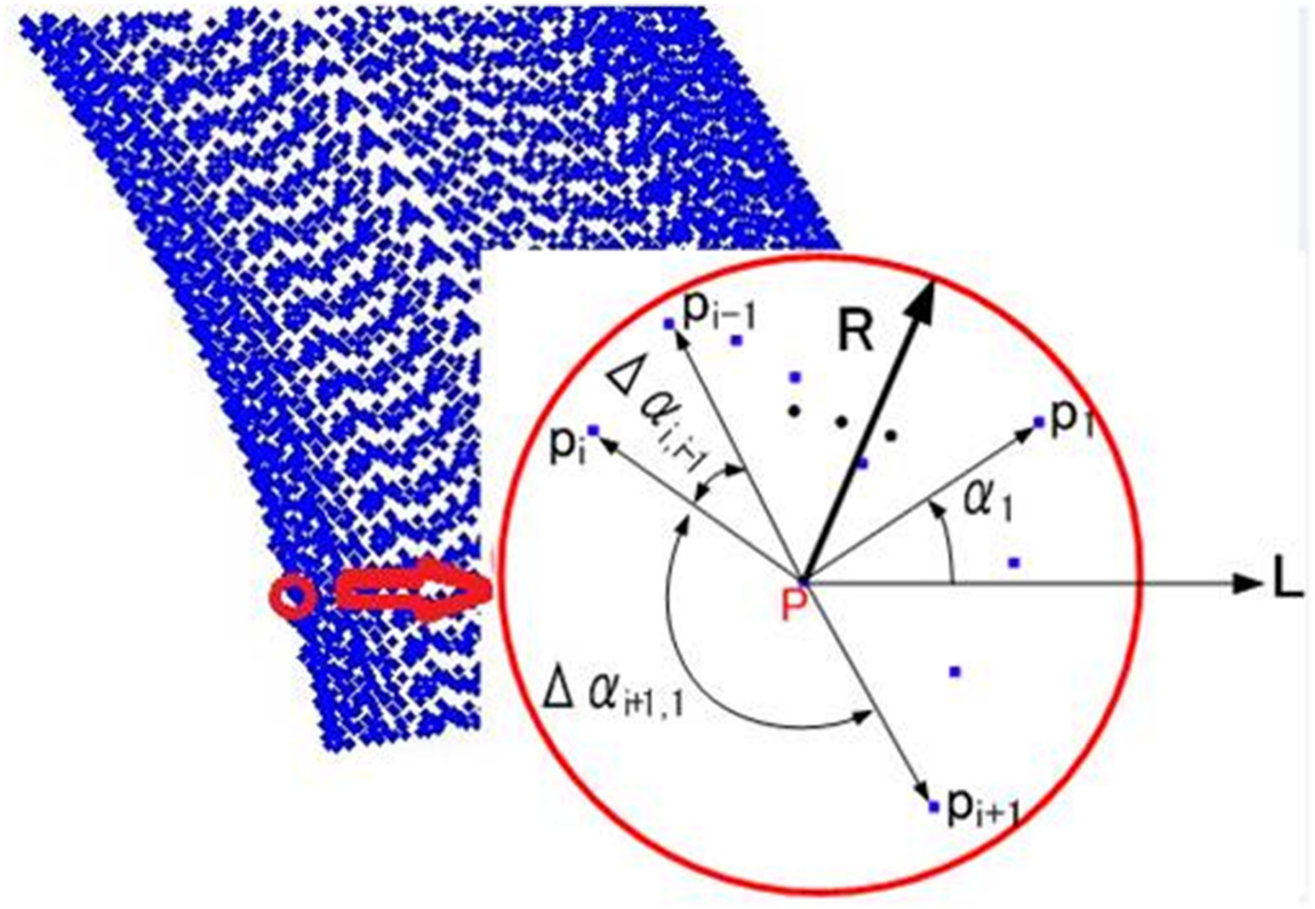
Extraction of boundary points using a moving window. All of the points within the moving window are considered to be neighboring points of point P. The polar angles of the neighboring points are computed relative to point P (e.g., α1). If point P is a boundary point, the difference between the consecutive polar angles of boundary points P_i_ and P_i + 1_ is much greater than the difference between the polar angles of point P_i_ and interior point P_i − 1_. Therefore, once the difference is greater than a predefined threshold, point P is labeled as a boundary point.

The tunnel point clouds are projected onto the XOY plane, from which we extract the boundary points of both sides of the tunnel. Therefore, the shape of the tunnel does not influence the extraction of the two parallel bounding lines on the XOY plane. An algorithm for boundary point extraction is proposed using a moving window. Fig 2 shows a circular window with a predefined radius that is centered at the point of interest P. All of the points within the window are considered as the neighboring points of point P. The polar angles of the neighboring points are computed relative to point P (e.g., *α*_1_). We then calculate the differences between consecutive polar angles. If point P is a boundary point, the difference Δ*α*_*i* + 1,*i*_ between boundary points P_*i*_ and P_*i* + 1_ is much larger than the difference Δ*α*_*i*,*i* − 1_ between boundary point P_*i*_ and interior point P_*i* − 1_. Therefore, once the difference is greater than a predefined threshold, point P is labeled as a boundary point.

#### Fitting of the bounding lines

The bounding lines of a tunnel usually contain segments that are straight lines, curves and transition curves, which are parameterized as follows:

Straight line model:

X=aY+b (13)

Transition curve model:

$$X=cY^{3}+dY^{2}+eY+f$$ (14)

Curve model:

$$X=gY^{2}+hY+k$$ (15)

where *a* and *b* are the parameters of a straight line, *c*, *d*, *e*, and *f* are the parameters of a transition curve, and *g*, *h*, and *k* are the parameters of a curve.

The bounding line fitting process includes the estimation of multiple models. To ensure the robustness of the fit, the RANSAC algorithm [15] is used to estimate the parameters of the three models. Instead of using as much data as possible to obtain an initial solution and attempting to eliminate the invalid data points, RANSAC uses as small an initial data set as is feasible and enlarges this set using consistent data when possible. The RANSAC paradigm contains three unspecified parameters: (1) the error tolerance, which is used to determine whether a point is compatible with the model; (2) the number of subsets to attempt; and (3) the threshold t, which is the number of compatible points and is used to determine that the correct model has been found. The determination of these three parameters is discussed in the introduction to RANSAC [15].

A statistical testing algorithm is proposed to implement a hypothesis testing process and automatically detect the initial models from the extracted boundary points to ensure that the proper model will be selected to fit each segment of the bounding line. The statistical test is implemented using the straight line, transition curve and curve models.

Because the mathematical models of the bounding line segments to be fitted are determinate, their parameters that are calculated by consecutive inlier sets are expected to converge in the direction of the optimal solution. Therefore, our strategy is based on a histogram to dynamically evaluate the convergence of the hypothesis models during the hypothesis testing process. Different convergent clusters of the hypothesis models will be presented in the histogram. We select the oldest model parameter set as the reference point for each convergent cluster of model parameter sets. The more the hypothesis models converge to a cluster, the more possible it is that the reference model of the cluster is correct. Therefore, we use the degree of convergence of a cluster to detect the initial models. The degree of convergence is a percentage that describes the number of models that converge to a model cluster. It is calculated by dividing the number of hypothesis models in the cluster by the total number of hypothesis models.

To determine the convergence of a newly computed model to a model cluster, we need to evaluate the deviation between the new model and the reference model of the cluster. We construct vectors with two (straight line), four (transition curve) and three (curve) dimensions for each set of model parameters. The Euclidian distances between different vectors are computed to describe the deviation between the new model and the reference model of the cluster. If the deviation is smaller than the predefined threshold, the number of FMs in the cluster is increased by one, and the degree of convergence is updated accordingly. If the new hypothesis model does not converge to any existing cluster, it will be regarded as a new cluster in the histogram.

In this method, the histogram of the candidate model parameter sets is updated during each iteration using the newly calculated hypothesis model parameter set. When the degree of convergence of a candidate model parameter set reaches a predefined threshold, the candidate model parameter set is detected as an initial model to fit the bounding line segment. If the degree of convergence fails to reach the threshold after a predefined number of iterations, we interpret that there is no such model.

**Fig 3:**
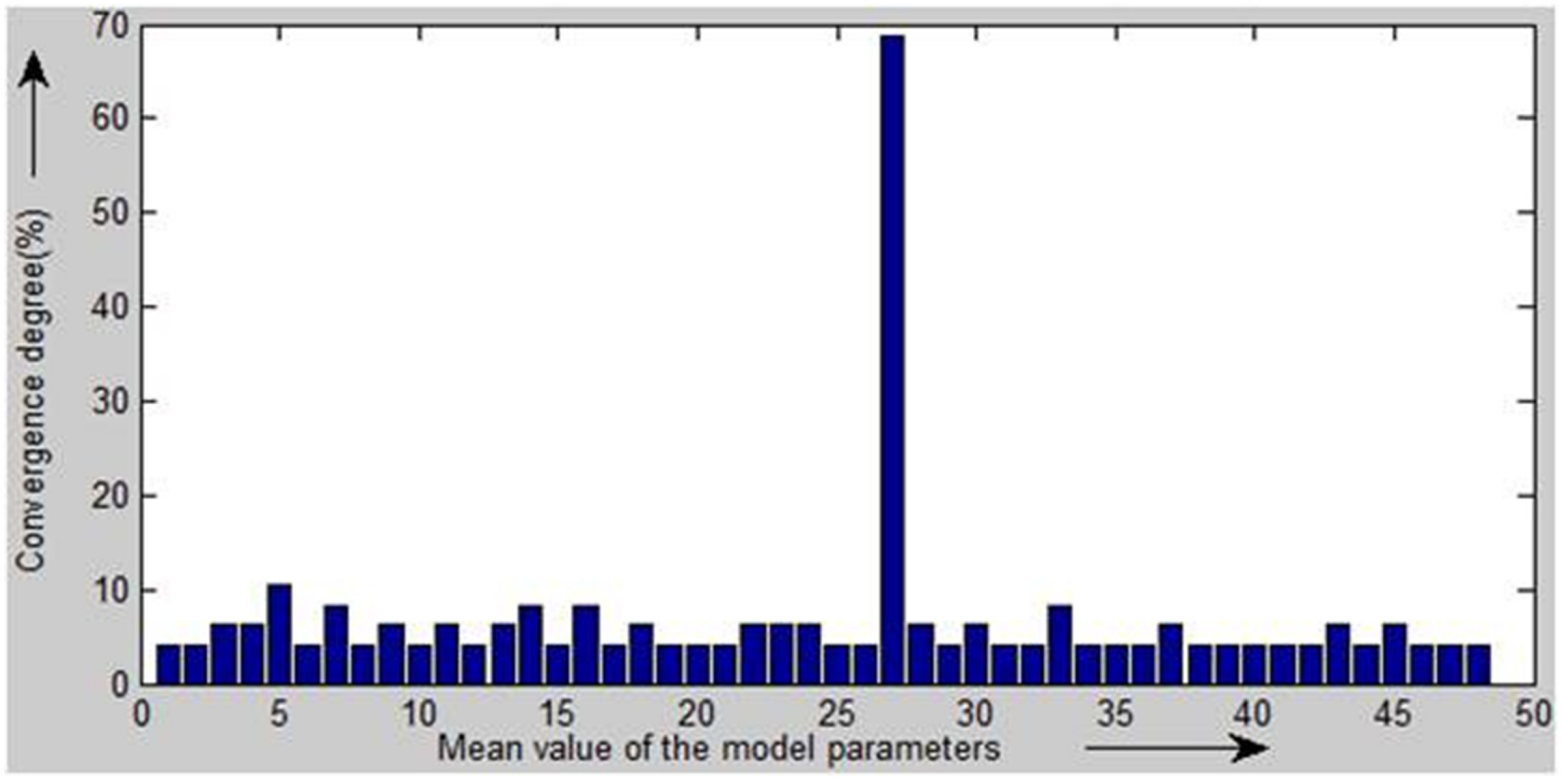
Histogram of hypothesis model parameters. The horizontal axis denotes the mean value of the model parameters, and the vertical axis represents the degree of convergence of each cell. A high degree of convergence for a parameter reflects a high probability of finding the initial model.

To visualize the statistical results, we illustrate them as a histogram (Fig 3). The horizontal axis denotes the mean value of the model parameters, and the vertical axis represents the degree of convergence of each cell. A high degree of convergence for a parameter reflects a high probability of finding the initial model.

After the initial model is detected, RANSAC is used to robustly estimate the optimized model parameters. Two, four, and three points are used to estimate the model parameters to fit a straight line, a transition curve and a curve, respectively. The criterion that is used to identify outliers is based on the deviations of the tested points from the fitted model. The inlier bounding points of a certain model are classified as a segment that is used in the following global optimization. The final optimal parameters are computed by a least-squares adjustment using the obtained inlier points.

#### Fitting of the central axis

**Fig 4:**
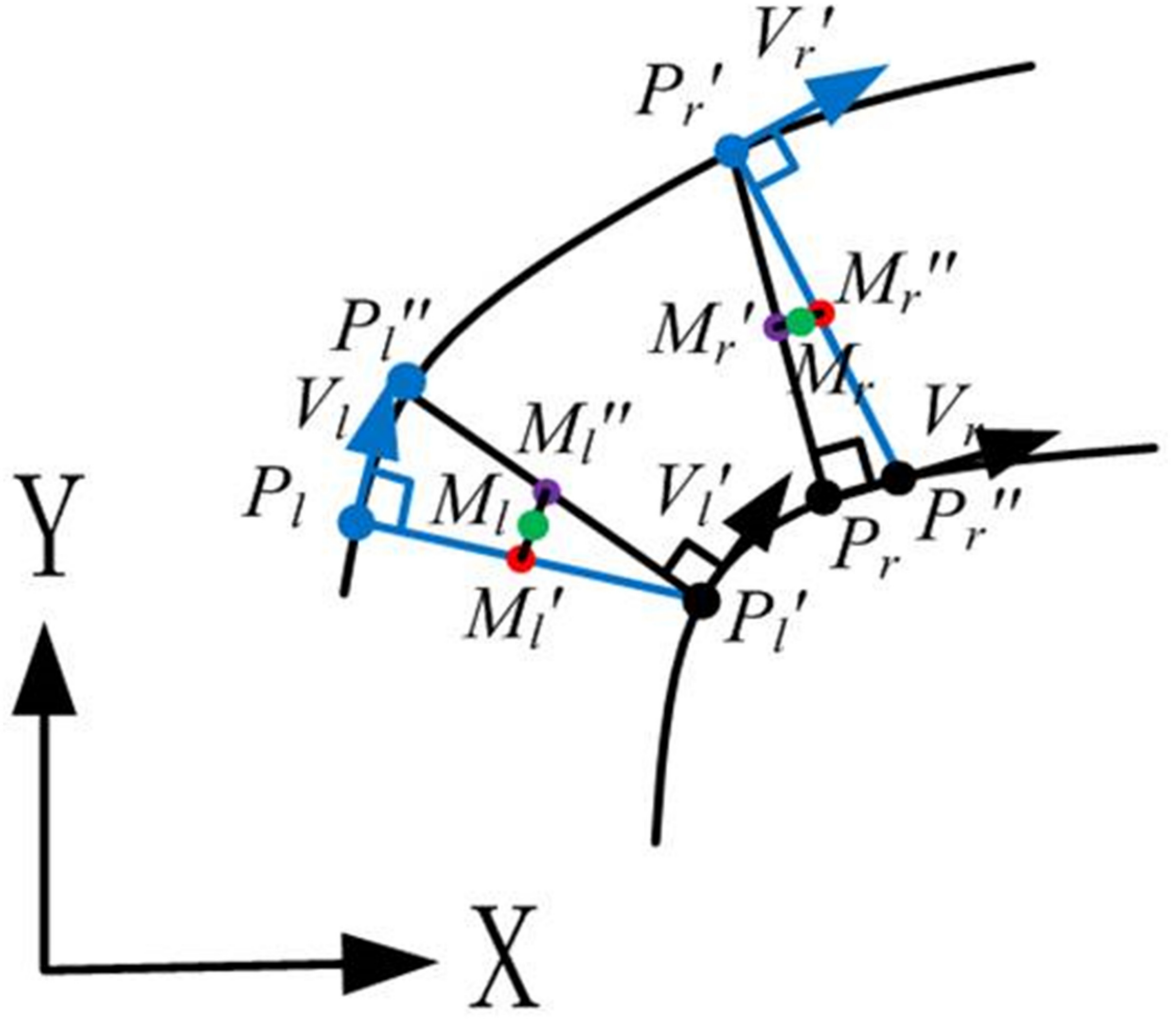
Determination of the central-axis point. To extract the central axis of the tunnel, the normal vector *V*_*l*_ of the left bounding curve at boundary point *P*_*l*_ is determined. A straight line orthogonal to the normal vector reaches the right bounding curve from *P*_*l*_ and generates point *P*_*l*_ *'*. Theoretically, the radial line from point *P*_*l*_ *'* that is orthogonal to *V*_*l*_ *'* reaches the left bounding curve at point *P*_*l*_, so the extracted central-axis point is the midpoint of line *P*_*l*_ *P*_*l*_ *'*. However, because the bounding curves are subject to errors that are generated from the fitting process, the radial that is orthogonal to *V*_*l*_ *'* produces point *P*_*l*_ *"* instead of point *P*_*l*_. *M*_*l*_ *'* and *M*_*l*_ *"* are the midpoints of *P*_*l*_ *P*_*l*_ *'* and *P*_*l*_ *' P*_*l*_ *"*, respectively. The extracted central-axis point is determined as *M*_*l*_, which is the average of points *M*_*l*_ *'* and *M*_*l*_ *"*. The same process is implemented from boundary point *P*_*r*_ on the right bounding curve to extract the point on the central axis as point *M*_*r*_. Based on the extracted central-axis points, the presented strategy to fit a bounding line is used to generate the central axis.

After fitting the bounding lines, the boundary points are evenly resampled. To extract the central axis of the tunnel, the normal vector *V*_*l*_ of the left bounding curve at boundary point *P*_*l*_ is determined (Fig 4). A straight line orthogonal to the normal vector reaches the right bounding curve from *P*_*l*_ and generates point *P*_*l*_ *'*. Theoretically, the radial line from point *P*_*l*_ *'* that is orthogonal to *V*_*l*_ *'* reaches the left bounding curve at point *P*_*l*_, so the extracted central-axis point is the midpoint of the line *P*_*l*_ *P*_*l*_ *'*. However, because the bounding curves are subject to errors that are generated from the fitting processes, the radial orthogonal to *V*_*l*_ *'* produces point *P*_*l*_ *"* instead of point *P*_*l*_. Fig 4 shows that *M*_*l*_ *'* and *M*_*l*_ *"* are the midpoints of *P*_*l*_ *P*_*l*_ *'* and *P*_*l*_ *' P*_*l*_ *"*, respectively. The extracted central-axis point is determined as *M*_*l*_, which is the average of points *M*_*l*_ *'* and *M*_*l*_ *"*. The same process is implemented from boundary point *P*_*r*_ on the right bounding curve to extract the point on the central axis as point *M*_*r*_. The presented strategy to fit a bounding line is used to generate the central axis based on the extracted central-axis points. Because the extraction of the central axis is implemented on the XOY plane, the height of the central axis is determined as the mid-height of the tunnel points.

### Global adjustment of the central axis using segment-wise fitting

**Fig 5:**
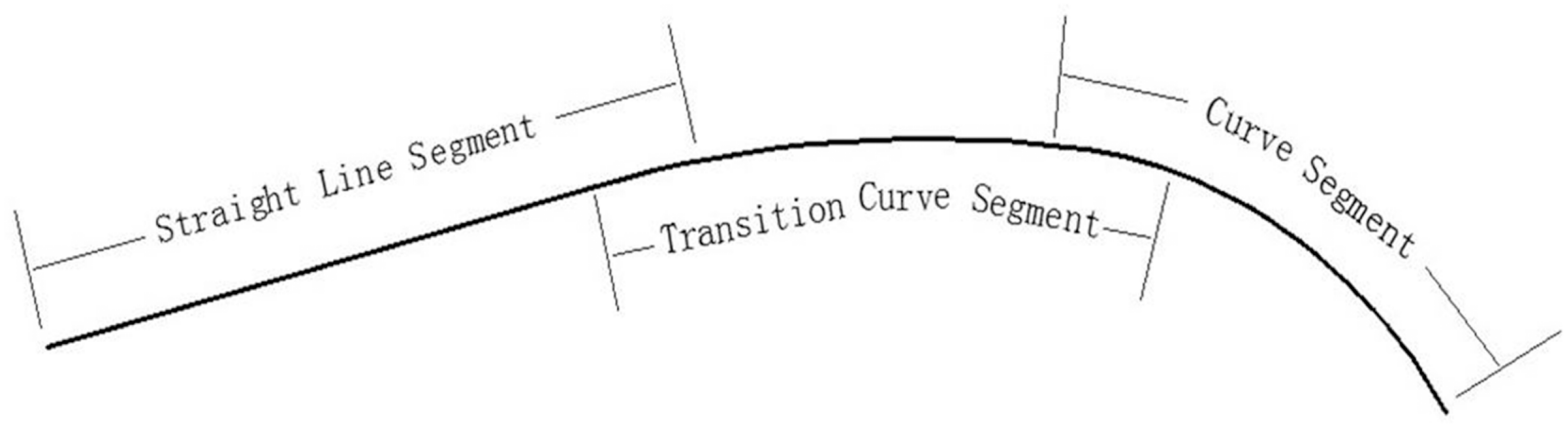
Segment-wise fitting. To maintain consistency between adjacent fitted models, the divided segments overlap each other slightly, and a global least-squares adjustment is developed to implement the multiple model fittings of all of the segments together by minimizing the deviations in the overlapping parts of adjacent fitted models.

Because the extraction of the segments of the bounding lines and the central axis on the XOY plane using the three models may be affected by noise in the tunnel points, there may be deviations in the overlapping parts of adjacent fitted models (Fig 5). Therefore, we propose a global extraction algorithm to minimize the deviations.

To maintain consistency between adjacent fitted models, the divided segments overlap each other by a small amount, and a global least-squares adjustment is developed to implement the multiple model fitting of all of the segments together by minimizing the deviations in the overlapping parts of adjacent fitted models. Using Eqs (13)–(15), the constraints are derived between a straight line, a transition curve and a curve, respectively, and are added to the adjustment model. For example, Eq (16) parameterizes the constraint between a straight line and a transition curve:

$$a_{i}Y+b_{i}=\left(c_{j}Y^{3}+d_{j}Y^{2}+e_{j}Y+f_{j}\right)=0$$ (16)

where *a*_*i*_ and *b*_*i*_ are the line parameters of segment *i*, *c*_*j*_, *d*_*j*_, *e*_*j*_, and *f*_*j*_ are the transition curve parameters of segment *j*, and *Y* is the Y coordinate of a point in the overlap region between segments *i* and *j*.

Eq (16) describes the constraint that the X coordinates computed by any Y coordinate in the overlap region between segments *i* and *j* using the model parameters of segments *i* and *j* are theoretically equal.

The coefficient matrix of the observation and constraint equations of the global least-squares adjustment is derived in Eq (17):

$$B=\left[\begin{array}{ccc} B_{l} & & \\ & B_{tc} & \\ & & B_{c}\\ & C & \end{array}\right]$$ (17)

where

$$\begin{array}{l} B_{l}=\left[\begin{array}{cccc} B_{l_{1}} & 0 & 0 & 0\\ 0 & B_{l_{2}} & 0 & 0\\ 0 & 0 & \cdots & 0\\ 0 & 0 & 0 & B_{l_{n1}} \end{array}\right],B_{c}=\left[\begin{array}{cccc} B_{c_{1}} & 0 & 0 & 0\\ 0 & B_{c_{2}} & 0 & 0\\ 0 & 0 & \cdots & 0\\ 0 & 0 & 0 & B_{c_{n2}} \end{array}\right],\\ B_{tc}=\left[\begin{array}{cccc} B_{tc_{1}} & 0 & 0 & 0\\ 0 & B_{tc_{2}} & 0 & 0\\ 0 & 0 & \cdots & 0\\ 0 & 0 & 0 & B_{tc_{n3}} \end{array}\right],B_{li}=\left[\begin{array}{cc} y_{l_{i,1}} & 1\\ y_{l_{i,2}} & 1\\ \vdots & 1\\ y_{l_{i,m}} & 1 \end{array}\right],B_{c_{i}}=\left[\begin{array}{ccc} y_{c_{i,1}}^{2} & y_{c_{i,1}} & 1\\ y_{c_{i,2}}^{2} & y_{c_{i,2}} & 1\\ \vdots & \vdots & \vdots \\ y_{c_{i,m}}^{2} & y_{c_{i,m}} & 1 \end{array}\right],and\\ B_{tc_{i}}=\left[\begin{array}{cccc} y_{tc_{i,1}}^{3} & y_{tc_{i,1}}^{2} & y_{tc_{i,1}}^{} & 1\\ y_{tc_{i,2}}^{3} & y_{tc_{i,2}}^{2} & y_{tc_{i,2}}^{} & 1\\ \vdots & \vdots & \vdots & \vdots \\ y_{tc_{i,m}}^{3} & y_{tc_{i,m}}^{2} & y_{tc_{i,m}}^{} & 1 \end{array}\right], \end{array}$$ (18)

which are derived from Eqs (13)–(15) for segment *i*, *m* denotes the number of the points on segment *i*, *n* = *n*1 + *n*2 + *n*3, and

$$C=\left[\begin{array}{ccccccc} C_{11} & C_{12} & 0 & 0 & 0 & 0 & 0\\ 0 & C_{22} & C_{23} & 0 & \vdots & \vdots & \vdots \\ \vdots & \vdots & \vdots & \ddots & 0 & 0 & 0\\ 0 & 0 & 0 & 0 & 0 & C_{\left(n-1)(n-1\right)} & C_{(n-1)n} \end{array}\right]$$ (19)

where

$$\begin{array}{l} C_{ij}=\left[\begin{array}{ccc} y_{i1,j(j+1)}^{2} & y_{i1,j(j+1)} & 1\\ y_{i2,j(j+1)}^{2} & y_{i2,j(j+1)} & 1\\ \vdots & \vdots & \vdots \\ y_{ik,j(j+1)}^{2} & y_{ik,j(j+1)} & 1 \end{array}\right],or\;C_{ij}=\left[\begin{array}{cccc} y_{i1,j(j+1)}^{3} & y_{i1,j(j+1)}^{3} & y_{i1,j(j+1)} & 1\\ y_{i2,j(j+1)}^{3} & y_{i1,j(j+1)}^{2} & y_{i2,j(j+1)} & 1\\ \vdots & \vdots & \vdots & \vdots \\ y_{ik,j(j+1)}^{3} & y_{ik,j(j+1)}^{2} & y_{ik,j(j+1)} & 1 \end{array}\right],or\\ C_{ij}=\left[\begin{array}{cc} y_{i1,j(j+1)} & 1\\ y_{i2,j(j+1)} & 1\\ \vdots & 1\\ y_{ik,j(j+1)} & 1 \end{array}\right],and\;C_{i(j+1)}=-C_{ij} \end{array}$$ (20)

which are derived from Eq (17) for the overlap region between segment *j* and segment *j* + 1. The form of *C*_*ij*_ depends on the models of the two overlapping segments. In the proposed global least-squares adjustment system, Eq (16) is used as a constraint equation and is weighted with a large value (e.g., 10) instead of with 1, as occurs in an observation equation. Based on the coefficient matrix B, we calculate the optimized parameters of the bounding line segments by following the least-squares strategy. After the bounding lines are fitted, the method presented in Section 3.1 is implemented to extract the central-axis points, which we use to generate the globally optimized central axis using the proposed global least-squares adjustment system.

### Observation model derived from the central-axis constraint

The extracted central axis comprises multiple segments that can be parameterized using Eqs (13)–(15). We derive the central-axis constraint by adding an additional observation model into the AEKF system.

**Fig 6:**
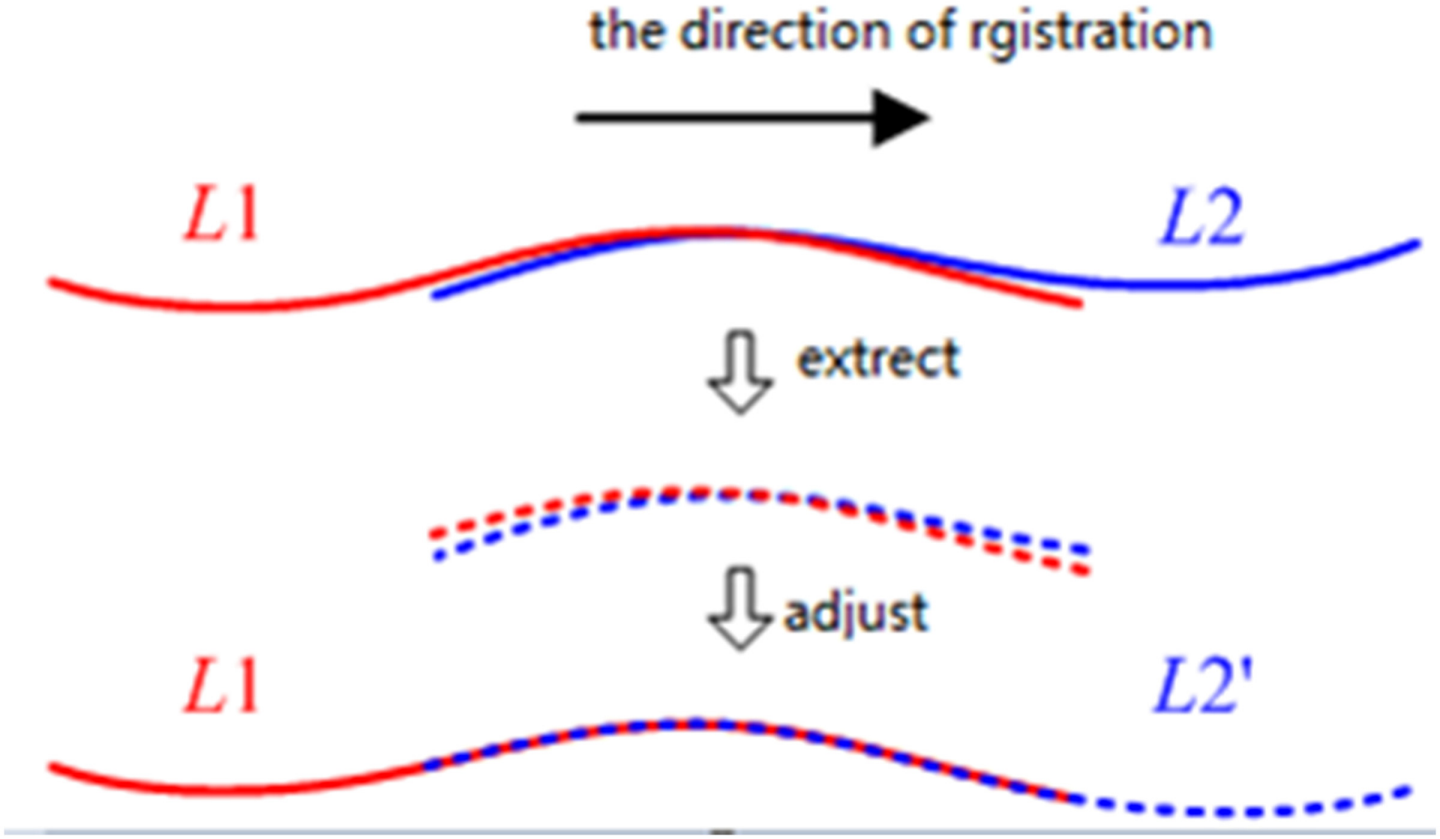
Central-axis constraint. We derive the central-axis constraint by adding an additional observation model to the AEKF system. The observation model is established to minimize the deviation that describes how the point on the axis segment in the analyzed scan does not fit the corresponding axis segment model that was determined in the fixed scan when the point is transformed into the global reference frame.

The observation model is established to minimize the deviation that describes how the point on the axis segment in the analyzed scan does not fit the corresponding axis segment model that was determined in the fixed scan (Fig 6), when the point is transformed into the global reference frame. Therefore, the observation value Z is computed as (for the straight line model):

$$Z_{A}=h_{A}\left(X_{m}\left(k\right),\text{…},X_{n}\left(k\right)\right)+\upsilon_{A}^{}\left(k\right)=\left[\begin{array}{ccc} 1 & -a & 0\\ 0 & 0 & 1 \end{array}\right]\left[\begin{array}{c} X_{i}{{}_{a}}^{\prime }\\ Y_{i}{{}_{a}}^{\prime }\\ Z_{i}{{}_{a}}^{\prime } \end{array}\right]-\left[\begin{array}{c} b\\ Z_{mid} \end{array}\right]+\upsilon \left(k\right)$$ (21)

where (*a*, *b*) represent the linear parameters, *Z*_*mid*_ denotes the middle height of the tunnel points in the overlap, *h*(.) is the observation model, and *υ*(*k*) denotes a variety of uncertainties in the scanning measurement and the transformation of coordinates, which is supposed to comply with the Gaussian distribution and is thus expressed as a white noise vector Ν (0, R)

$$\left[\begin{array}{c} X_{i}{{}_{a}}^{\prime }\\ Y_{i}{{}_{a}}^{\prime }\\ Z_{i}{{}_{a}}^{\prime } \end{array}\right]=R_{m}\cdots R_{n}\left[\begin{array}{c} X_{i}{}_{a}\\ Y_{i}{}_{a}\\ Z_{i}{}_{a} \end{array}\right]+\left[\begin{array}{c} T_{m,X}\\ T_{m,Y}\\ T_{m,Z} \end{array}\right]+R_{m}\left[\begin{array}{c} T_{m+1,X}\\ T_{m+1,Y}\\ T_{m+1,Z} \end{array}\right]+\cdots +R_{m}\cdots R_{n-1}\left[\begin{array}{c} T_{n,X}\\ T_{n,Y}\\ T_{n,Z} \end{array}\right]$$ (22)

The observation model is linearized as:

$$Z_{A}=h_{A}{}^{0}(X_{m}(k),\text{…},X_{n}(k))+\nabla h_{A}*\left[\begin{array}{c} \Delta X_{m}(k)\\ \vdots \\ \Delta X_{n}(k) \end{array}\right]+\upsilon \left(k\right)$$ (23)

where ∇*h*_*A*_ is the Jacobian matrix derived from Eqs (21) and (22)).

Because the observation model is established to minimize the deviation that describes how the point on the axis segment in the analyzed scan does not fit the corresponding axis segment model that was determined in the fixed scan, the selection of the number of observation equations is flexible in the proposed algorithm. The more observation equations that are added to the AEKF system, the greater the contribution that the constraint has on the registration. Thus, a constraint that is derived from a low-quality central-axis segment is assigned a small number of observation equations in our algorithm.

Therefore, the observation model of the AEKF system is modified as:

$$Z=\left[\begin{array}{c} Z_{P}\\ Z_{A} \end{array}\right]$$ (24)

The RTPs of all of the pair-wise registrations are then optimized by minimizing both the differences between the corresponding 3D point pairs and the point-to-model deviation, which is expected to improve the robustness and accuracy of the proposed global registration approach. The improvement can be attributed to the multiple overlaps of the central axis among the scans, while we are most likely able to find the tie points only from the overlaps between two consecutive scans.

## Experimental Results

**Fig 7:**
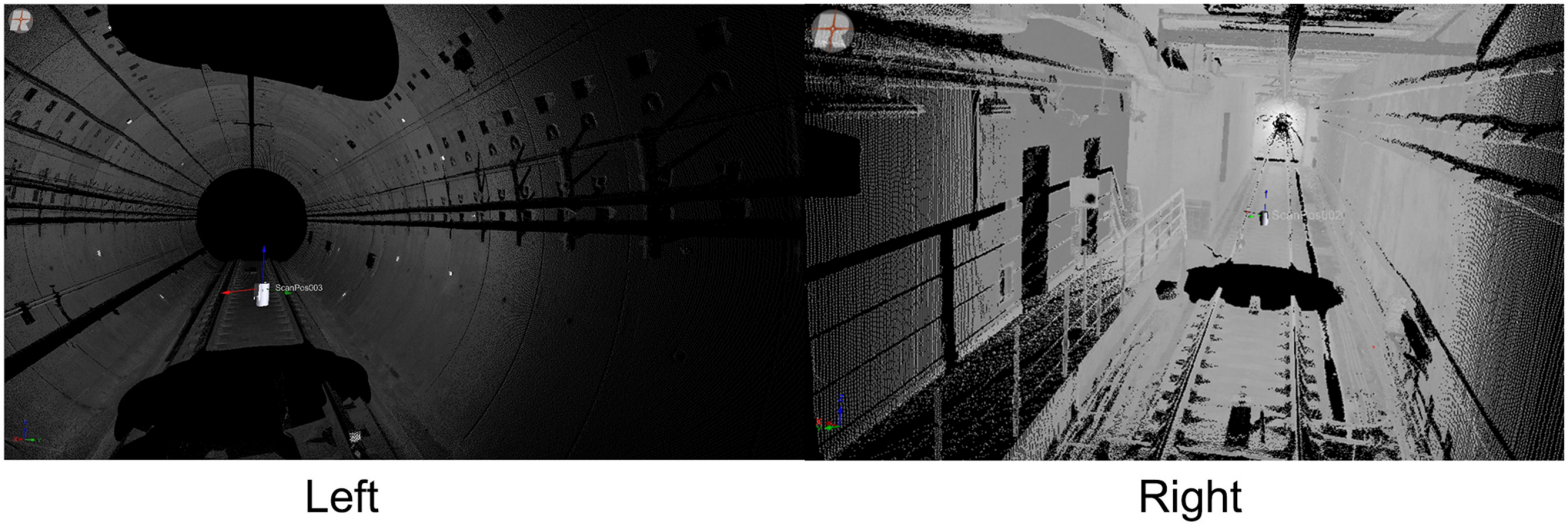
Experimental dataset. The proposed approach was tested on real datasets that were acquired by a RIEGL LMS VZ-400 laser scanner in a subway tunnel in Shanghai, China. Twelve scans were captured; the scans had an average shift of 10 m between their centers and cover tunnel segments with different shapes for a distance of 450 m.

The proposed approach was tested on real datasets (Fig 7) that were acquired by a RIEGL LMS VZ-400 laser scanner in a subway tunnel in Shanghai, China. Twelve scans were captured with an average shift of 10 m between the scanning centers. The scans cover tunnel segments with different shapes for a distance of 450 m. The scan’s angular resolution was 0.046°, and the range accuracy was ±5 mm.

### Pair-wise registration

**Fig 8:**
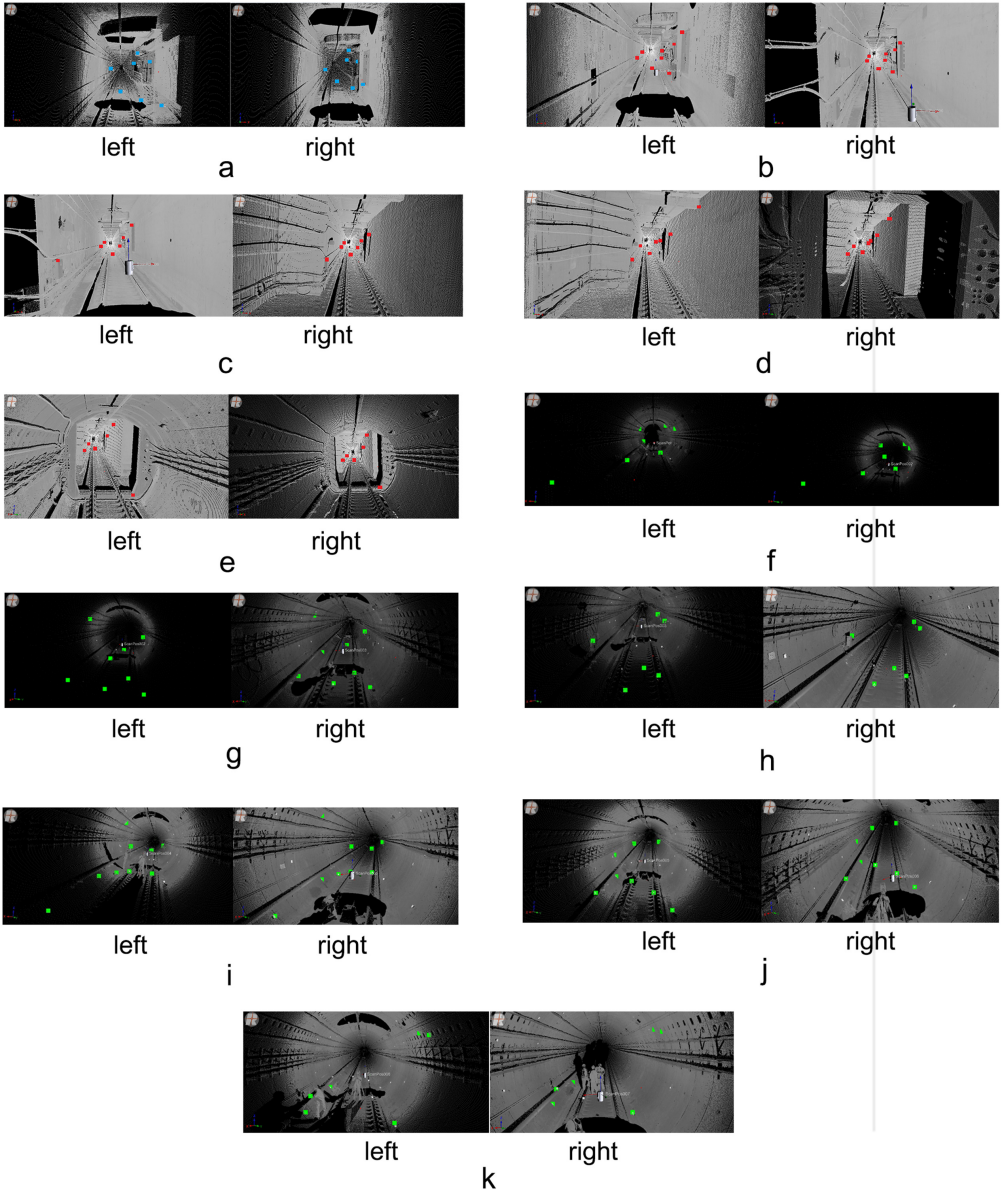
Tie points selected for registration. **(a)** Tie points (7 pairs) between Scans 1 and 2; **(b)** Tie points (8 pairs) between Scans 2 and 3; **(c)** Tie points (7 pairs) between Scans 3 and 4; **(d)** Tie points (7 pairs) between Scans 4 and 5; **(e)** Tie points (6 pairs) between Scans 5 and 6; **(f)** Tie points (7 pairs) between Scans 6 and 7; **(g)** Tie points (8 pairs) between Scans 7 and 8; **(h)** Tie points (6 pairs) between Scans 8 and 9; **(i)** Tie points (9 pairs) between Scans 9 and 10; **(j)** Tie points (8 pairs) between Scans 10 and 11; **(k)** Tie points (6 pairs) between Scans 11 and 12.

Fig 8 shows the tie points (high-reflectivity targets) between consecutive scans. Based on these tie points, the pair-wise registrations were implemented using RiSCAN PRO from RIEGL. Table 1 lists the accuracies of the pair-wise registrations; the average accuracy is 0.021 m.

**Table 1:** Accuracies of the pair-wise registrations.

Registration	Number of tie points	Accuracy/m	Registration	Number of tie points	Accuracy/m
1–2	7	0.0259	7–8	8	0.0174
2–3	8	0.0182	8–9	6	0.0208
3–4	7	0.0154	9–10	9	0.0237
4–5	7	0.0292	10–11	8	0.0170
5–6	6	0.0183	11–12	6	0.0249
6–7	7	0.0184			

**Fig 9:**
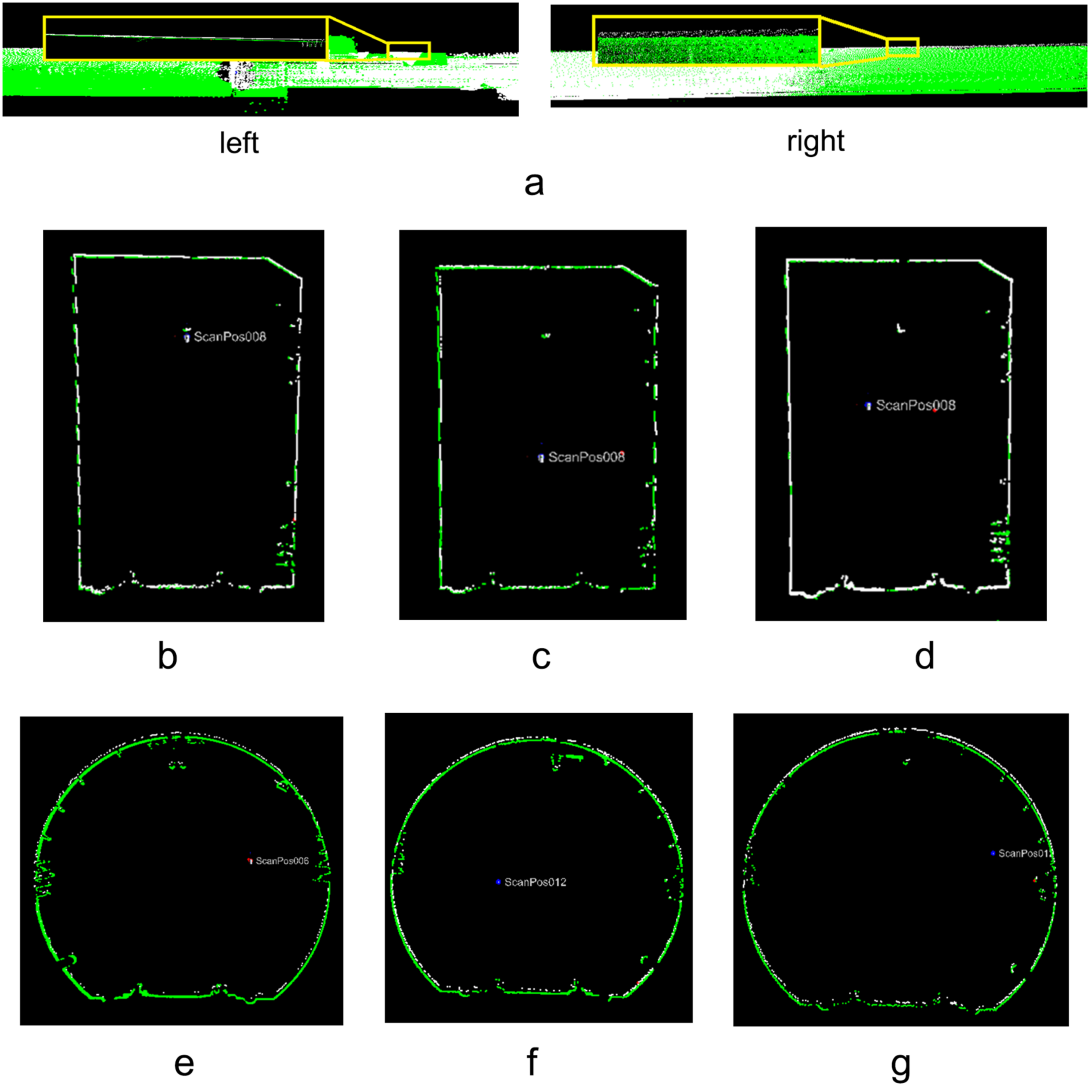
Error accumulation of pair-wise registration. (**a**) Overview; (**b**) Scans 1 and 4; (**c**) Scans 2 and 5; (**d**) Scans 3 and 6; (**e**) Scans 7 and 10; (**f**) Scans 8 and 11; (**g**) Scans 9 and 12.

As shown in Fig 7, the tunnel has a tubular shape, so the average length of the areas covered by the tie points is only 30 m, while the mean length of the tunnel segment that is captured by one scan is 150 m. Therefore, the accumulation of registration error is inevitable (Fig 9a). To estimate the error accumulation, Fig 9b–9g illustrate cross sections that were extracted at the same position from scans 1 and 4, scans 2 and 5, scans 3 and 6, scans 7 and 10, scans 8 and 11, scans 8 and 12, respectively, which were transformed into the same coordinate system using the pair-wise registration results. The distinct deviations between the two cross sections in Fig 9b–9g are due to the accumulation of the pair-wise registration errors.

Twenty check point pairs were selected from the two cross sections in Fig 9b–9g. Table 2 list the differences. The average deviation is 0.0463 m, which is larger than the average value of 0.022 m in Table 1.

**Table 2:** Deviations between check point pairs (Scans 1 and 4 to Scans 9 and 12).

	ID	Deviation/m	ID	Deviation/m	ID	Deviation/m	ID	Deviation/m
Scans 1 and 4	1	0.025	6	0.033	11	0.027	16	0.026
	2	0.031	7	0.019	12	0.044	17	0.009
	3	0.033	8	0.018	13	0.055	18	0.018
	4	0.025	9	0.061	14	0.039	19	0.031
	5	0.024	10	0.029	15	0.027	20	0.018
	RMSE/m							0.032
Scans 2 and 5	1	0.032	6	0.02	11	0.038	16	0.016
	2	0.034	7	0.013	12	0.032	17	0.033
	3	0.036	8	0.034	13	0.035	18	0.019
	4	0.039	9	0.04	14	0.037	19	0.047
	5	0.035	10	0.035	15	0.0036	20	0.043
	RMSE/m							0.0328
Scans 3 and 6	1	0.072	6	0.071	11	0.044	16	0.024
	2	0.067	7	0.057	12	0.039	17	0.034
	3	0.064	8	0.053	13	0.013	18	0.035
	4	0.066	9	0.038	14	0.039	19	0.019
	5	0.059	10	0.01	15	0.042	20	0.019
	RMSE/m							0.0473
Scans 7 and 10	1	0.069	6	0.061	11	0.015	16	0.046
	2	0.06	7	0.048	12	0.042	17	0.037
	3	0.071	8	0.037	13	0.076	18	0.03
	4	0.065	9	0.027	14	0.049	19	0.026
	5	0.061	10	0.008	15	0.04	20	0.034
	RMSE/m							0.0487
Scans 8 and 11	1	0.074	6	0.082	11	0.048	16	0.1
	2	0.078	7	0.063	12	0.059	17	0.046
	3	0.077	8	0.044	13	0.072	18	0.079
	4	0.115	9	0.05	14	0.092	19	0.038
	5	0.099	10	0.035	15	0.067	20	0.034
	RMSE/m							0.0713
Scans 9 and 12	1	0.093	6	0.081	11	0.046	16	0.0720
	2	0.082	7	0.086	12	0.038	17	0.0900
	3	0.075	8	0.077	13	0.018	18	0.0750
	4	0.073	9	0.08	14	0.064	19	0.0770
	5	0.02	10	0.074	15	0.08	20	0.0710
	RMSE/m							0.0717

As proposed in Section 2, we implemented the augmented extended Kalman filter to eliminate the error accumulation that was caused by the pair-wise registration to accurately estimate the rigid transformation parameters.

### Global registration using the augmented extended Kalman filter

**Fig 10:**
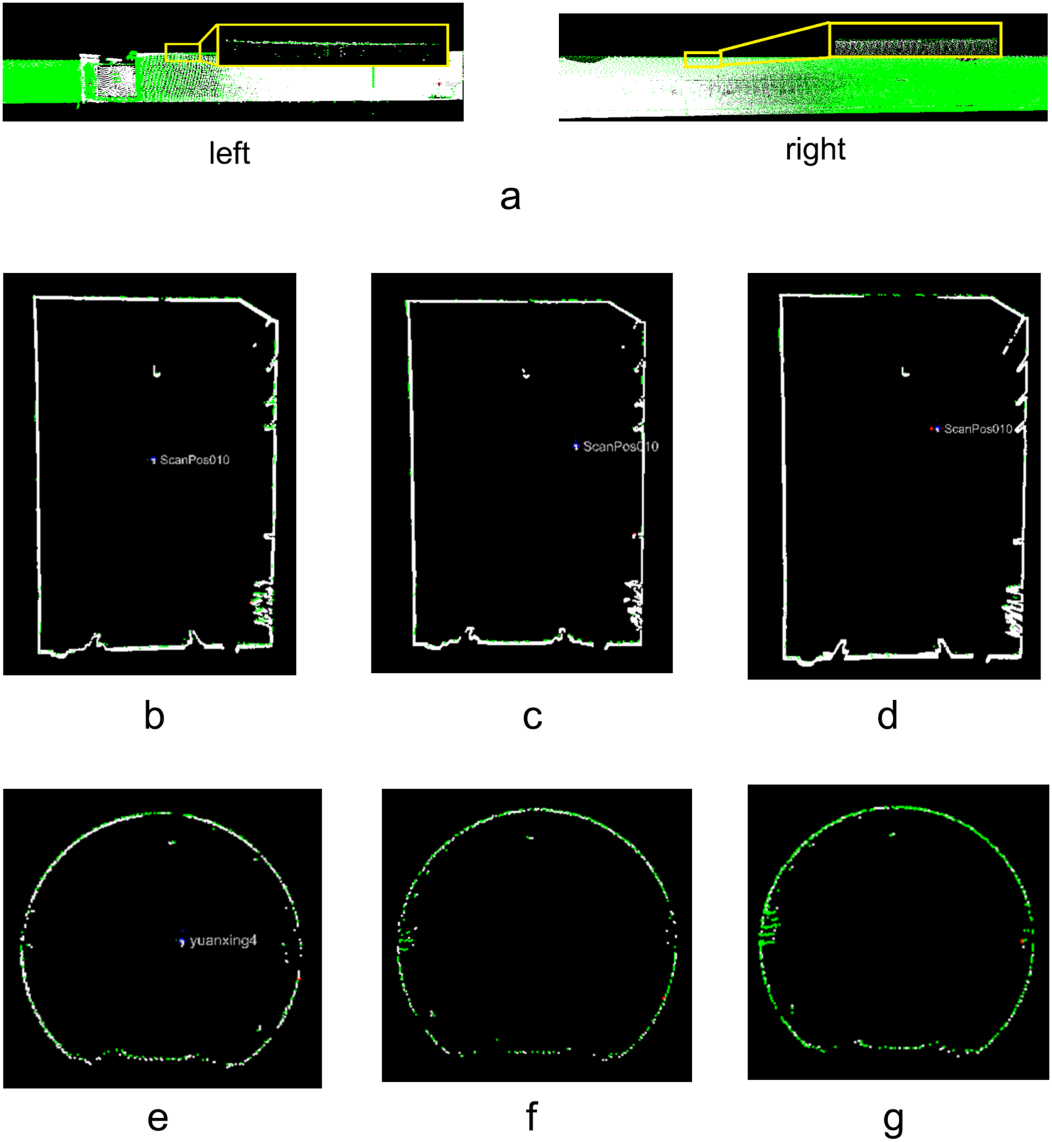
Global registration results (AEKF). (**a**) Overview; (**b**) Scans 1 and 4; (**c**) Scans 2 and 5; (**d**) Scans 3 and 6; (**e**) Scans 7 and 10; (**f**) Scans 8 and 11; (**g**) Scans 9 and 12.

The pair-wise registration results were used to construct the augmented extended Kalman filter system with which the global registration was implemented. To compare the registration accuracies, cross sections were also extracted at the same positions from scans 1 and 4, scans 2 and 5, scans 3 and 6, scans 7 and 10, scans 8 and 11, and scans 8 and 12 (Fig 10). Fig 10 shows that the deviations between the two cross sections are less than the deviations shown in Fig 9; the average deviation computed from the results in Table 3 decreases to 0.0301 m.

**Table 3:** Deviations between check point pairs (AEKF).

	ID	Deviation/m	ID	Deviation/m	ID	Deviation/m	ID	Deviation/m
Scans 1 and 4	1	0.038	6	0.028	11	0.03	16	0.0290
	2	0.041	7	0.026	12	0.026	17	0.0080
	3	0.038	8	0.031	13	0.027	18	0.0300
	4	0.027	9	0.027	14	0.027	19	0.0300
	5	0.025	10	0.027	15	0.017	20	0.0290
								0.0289
Scans 2 and 5	1	0.035	6	0.006	11	0.006	16	0.0090
	2	0.024	7	0.02	12	0.014	17	0.0110
	3	0.038	8	0.027	13	0.09	18	0.0040
	4	0.015	9	0.014	14	0.01	19	0.0200
	5	0.044	10	0.015	15	0.014	20	0.0230
								0.0290
Scans 3 and 6	1	0.05	6	0.029	11	0.028	16	0.0120
	2	0.013	7	0.032	12	0.036	17	0.0060
	3	0.016	8	0.015	13	0.022	18	0.0410
	4	0.041	9	0.027	14	0.026	19	0.0300
	5	0.03	10	0.017	15	0.026	20	0.0170
								0.0279
Scans 7 and 10	1	0.051	6	0.048	11	0.02	16	0.0370
	2	0.039	7	0.032	12	0.066	17	0.0290
	3	0.031	8	0.03	13	0.043	18	0.0320
	4	0.026	9	0.022	14	0.025	19	0.0070
	5	0.038	10	0.023	15	0.045	20	0.0150
								0.0355
Scans 8 and 11	1	0.031	6	0.008	11	0.014	16	0.0260
	2	0.042	7	0.025	12	0.015	17	0.0390
	3	0.023	8	0.032	13	0.033	18	0.0400
	4	0.047	9	0.022	14	0.034	19	0.0310
	5	0.036	10	0.019	15	0.049	20	0.0230
								0.0314
Scans 9 and 12	1	0.021	6	0.034	11	0.012	16	0.0230
	2	0.024	7	0.027	12	0.059	17	0.0250
	3	0.022	8	0.03	13	0.028	18	0.0210
	4	0.024	9	0.041	14	0.032	19	0.0330
	5	0.034	10	0.03	15	0.013	20	0.0230
								0.0295

Although the accumulation of errors of the pair-wise registration was reduced somewhat, the AEKF system was still implemented based on tie points that were shown to have a limited distribution. Therefore, as presented in Section 3, the constraint derived from the overlapping axes was introduced into the AEKF system to globally optimize the transformation parameters.

### Augmented extended Kalman filter with the central-axis constraint

To derive the central-axis constraint, the central-axis segments were first extracted from the twelve scans.

#### Fitting of the central axis based on the 2D projection

**Fig 11:**
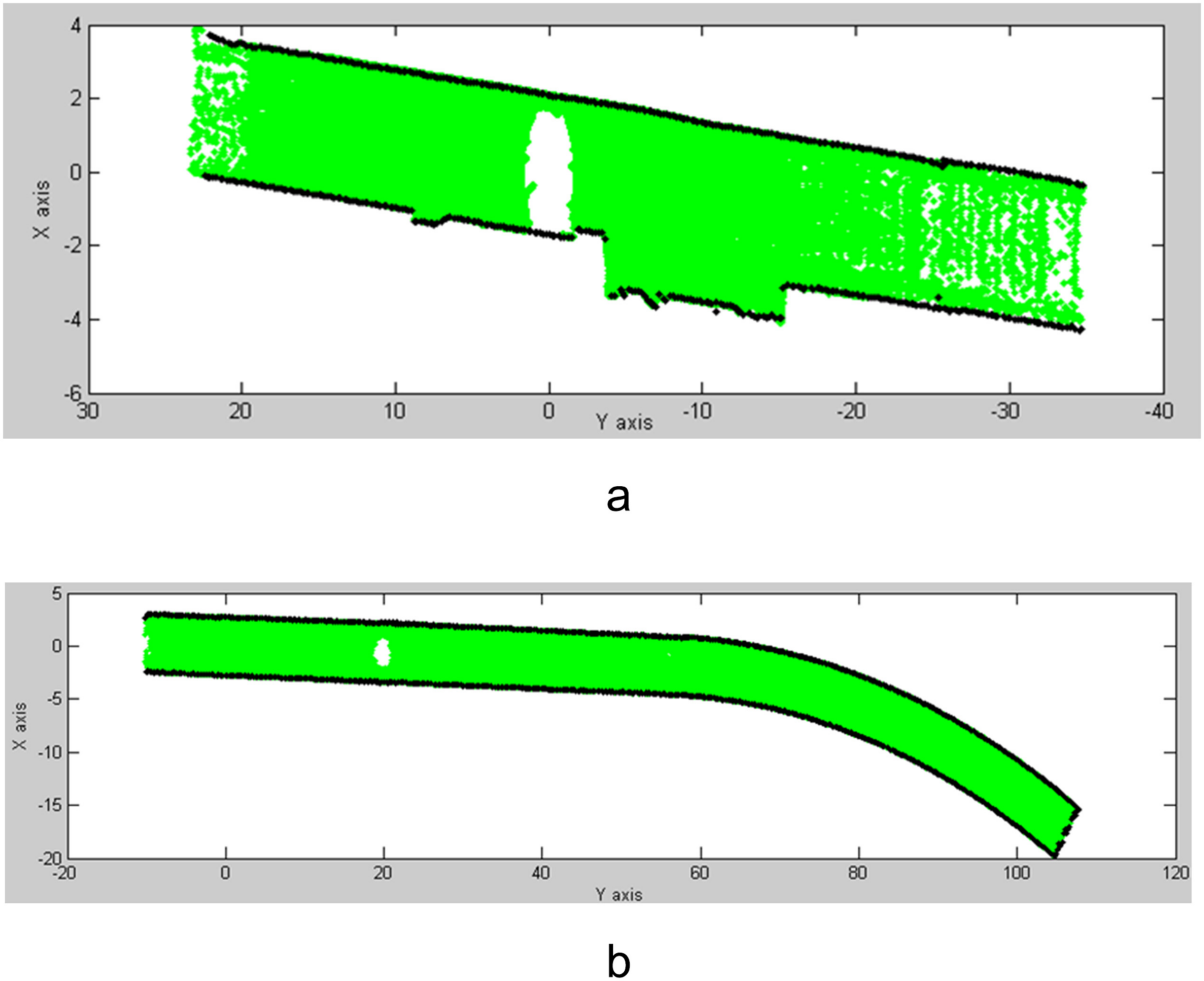
2D projections of the tunnel points onto the XOY plane (Scans 1 and 12). (a) 2D projection of Scan 1; (b) 2D projection of Scan 12.

As proposed in Section 3.1, the tunnel points of the dataset (e.g., scans 1 and 12) were projected onto the XOY plane as shown in Fig 11. The boundary points were extracted and are shown by the dark dots. Fig 11a shows that noise points are present due to the supplementary structures near the subway station.

**Fig 12:**
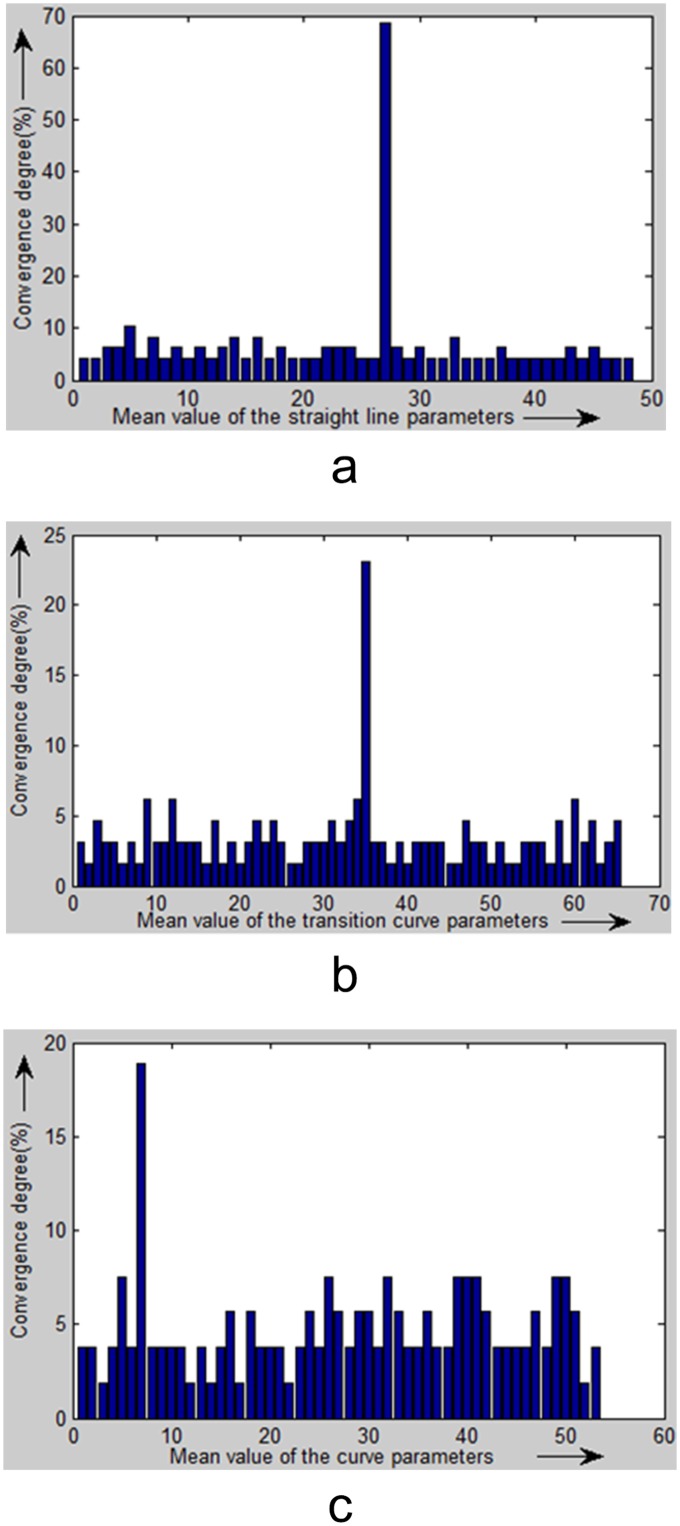
Statistical test results of the optimized BaySAC algorithm. (a) Straight line; (b) Transition curve; (c) Curve.

The bounding lines of a tunnel may comprise segments of straight lines, curves and transition curves. The proposed statistical testing algorithm was implemented using the three models to automatically detect the corresponding initial model from the extracted boundary points. Fig 12 shows the peaks in the histograms for the straight lines, transition curves and curves of the extracted boundary points that were fit using the three models.

**Fig 13:**
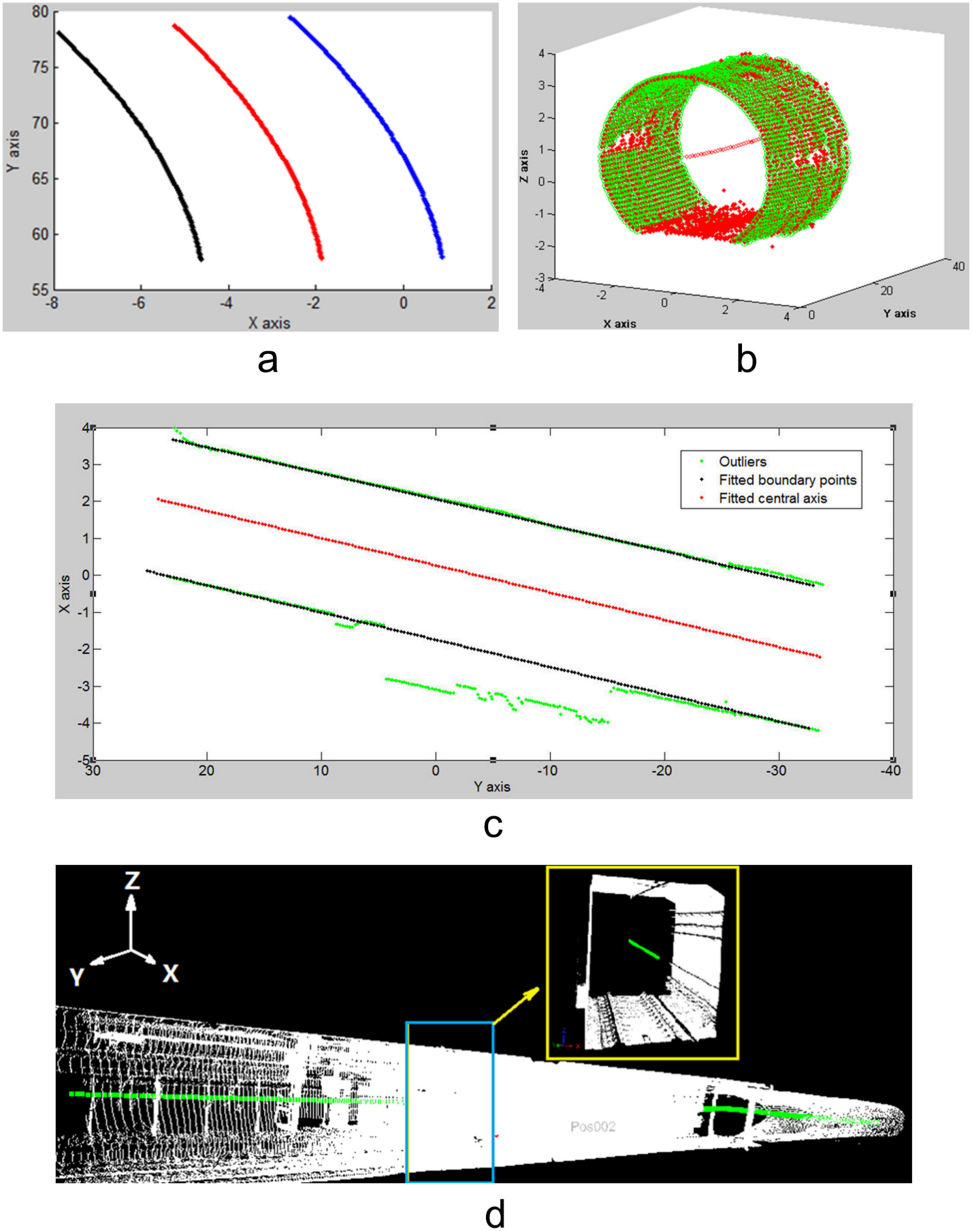
Fitting of the tunnel axis (straight line and transition curve). (**a**) Central-axis extraction (Scan 12); (**b**) extracted 3D central axis (Scan 12); (**c**) central-axis extraction (Scan 1); (**d**) extracted 3D central axis (Scan 1).

Based on the detected initial models (e.g., the straight line and transition curve), we fit the bounding lines using RANSAC (Fig 13a and 13c). In Fig 13c, the noise points shown in Fig 11a were eliminated (highlighted in green) by RANSAC. The boundary points were evenly resampled in terms of the fitted model parameters. The method presented in Section 3.1 was implemented using these fitted parameters to extract the central-axis points (Fig 13a and 13c). Fig 13b and 13d show 3D views of the central axes that were generated from the central-axis points.

**Fig 14:**
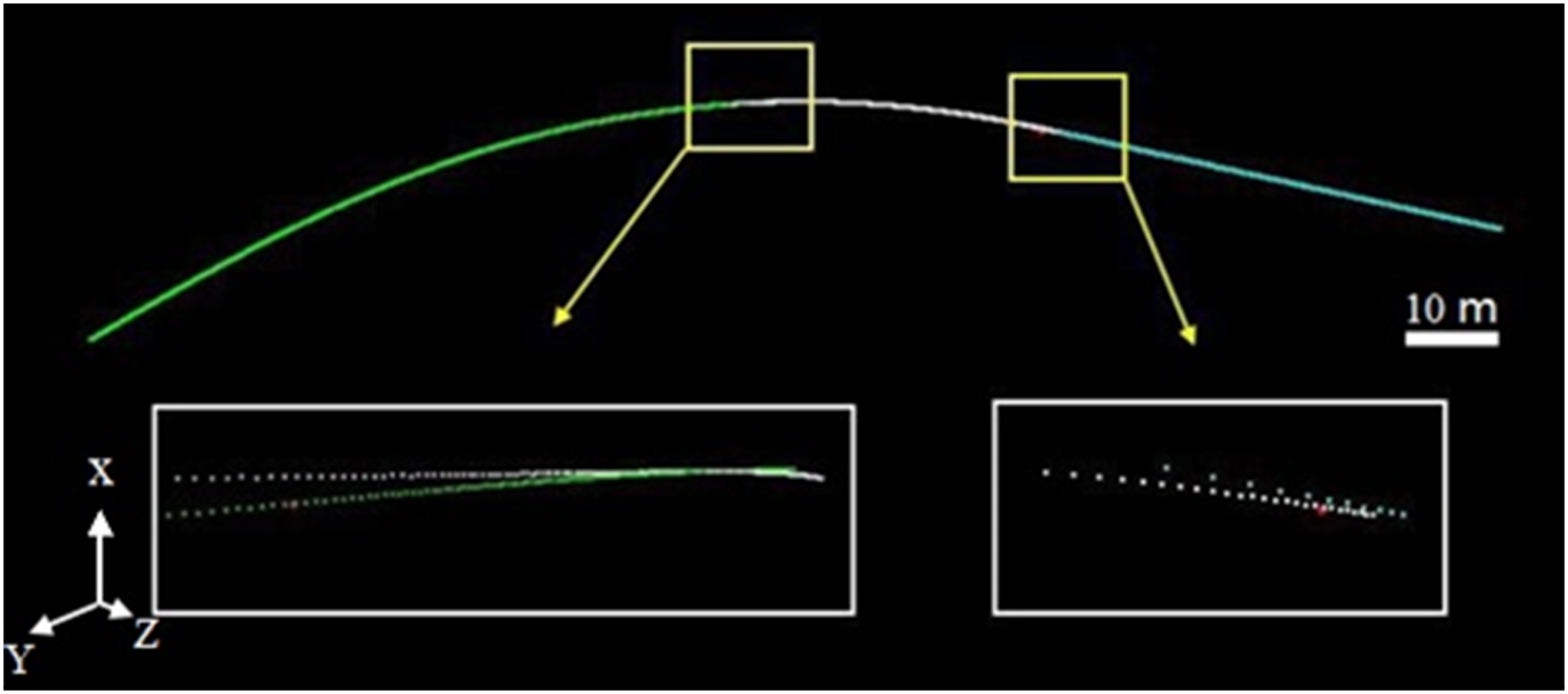
Extracted central axis. The central axis that was fit from the tunnel points consists of three segments. The yellow box on the left highlights the overlap between the curve and the transition curve, while the box on the right shows the overlap between the transition curve and the straight line. To test the fitting accuracy, we set 24 Y coordinates along the central axis within the overlap zones highlighted in the two yellow boxes. Their corresponding points on the two adjacent segments were computed. Large deviations with an RMSE of 26 mm (detailed views) are present within the overlap zones between the segments due to the noise in the tunnel point dataset.

As shown in Fig 14, the central axis that was fit from the tunnel points consists of three segments. The yellow box on the left highlights the overlap between the curve and the transition curve, while the box on the right shows the overlap between the transition curve and the straight line. To test the fitting accuracy, we set 24 Y coordinates along the central axis within the overlap zones highlighted in the two yellow boxes and computed their corresponding points on the two adjacent segments. The deviations between the corresponding points were then calculated and are shown in Table 4. Large deviations with an RMSE of 26 mm (detailed views) are present within the overlap zones between the segments due to the noise in the tunnel point dataset.

**Table 4:** Comparison of the fitting accuracies.

ID	Deviations after Global Extraction (m)	Deviations before Global Extraction(m)	ID	Deviations after Global Extraction (m)	Deviations before Global Extraction (m)
1	0.005	0.035	13	0.001	0.056
2	−0.003	−0.026	14	0.004	0.039
3	0.002	0.019	15	−0.005	0.022
4	0.003	0.016	16	0.003	0.047
5	−0.005	0.002	17	0.001	−0.032
6	0.001	0.001	18	0.004	0.03
7	−0.003	−0.007	19	0.001	0.038
8	0.002	0.003	20	−0.007	−0.015
9	−0.001	0.008	21	−0.001	0.035
10	−0.003	0.004	22	0.003	0.002
11	0.0013	0.01	23	0.001	−0.025
12	−0.002	−0.035	24	0.002	0.015
RMSE				0.002	0.026

#### Global extraction of the central axis using segment-wise fitting

**Fig 15:**
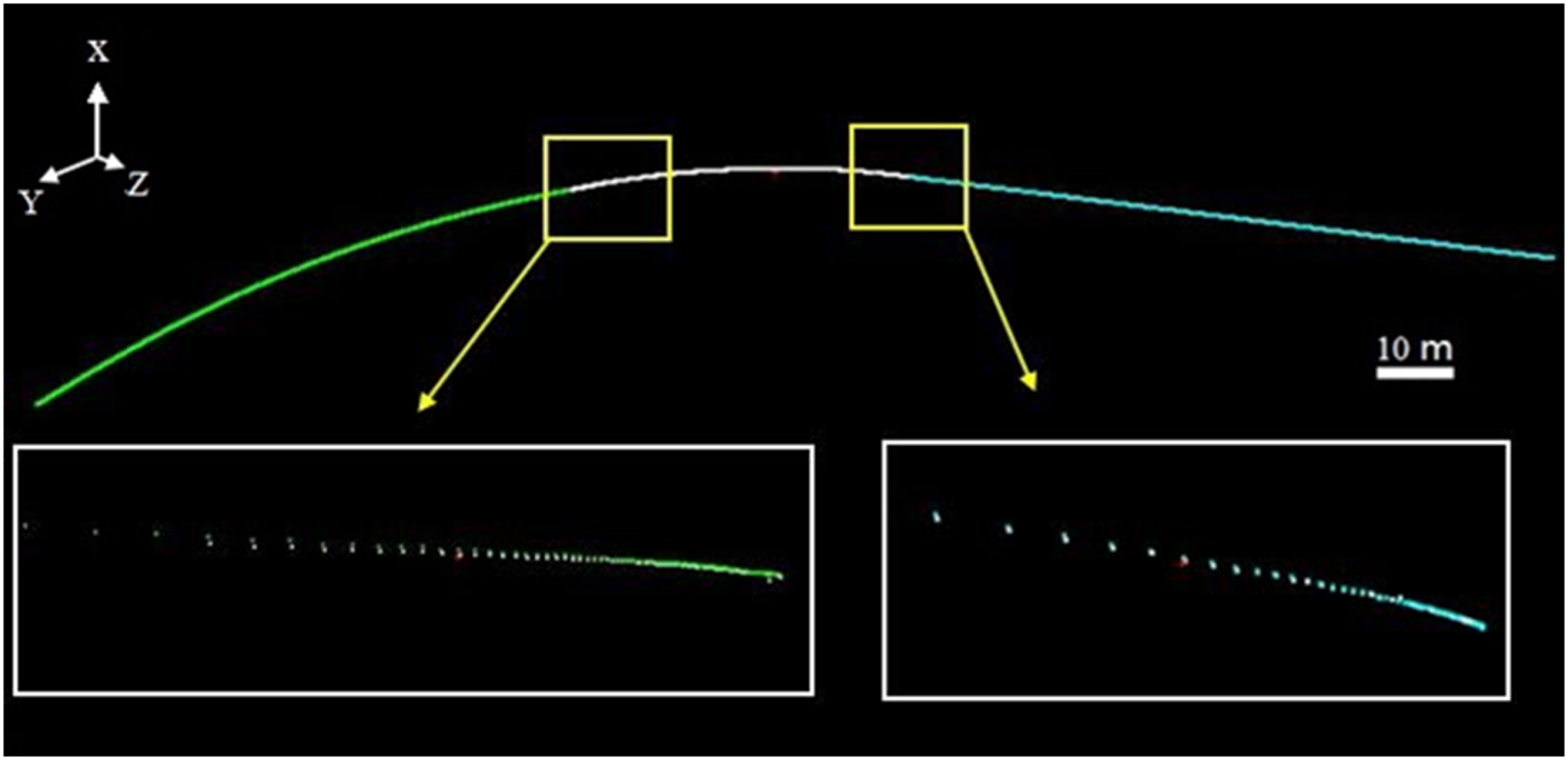
Central-axis fitting with global least squares adjustment. To optimize the extraction results, the proposed global least squares adjustment was implemented to minimize the deviations in the overlap zones between the adjacent fitted models. Fig 15 shows that the differences shown in Fig 14 were reduced significantly, and a globally optimized central axis was extracted.

To optimize the extraction results, the global least squares adjustment proposed in Section 3.2 was implemented to minimize the deviations in the overlap zones between the adjacent fitted models. Fig 15 shows that the differences in Fig 14 were reduced considerably, and a globally optimized central axis was extracted.

To test the fitting accuracy, we set 24 Y coordinates along the central axis within the overlap zones highlighted in the two yellow boxes. Their corresponding points on the two adjacent segments were computed. The deviations between the corresponding points were then calculated and are shown in Table 4. The RMSE of the deviations was reduced from 26 mm to 2 mm by the global extraction process.

#### Augmented extended Kalman filter with segment-wise axes

**Fig 16:**
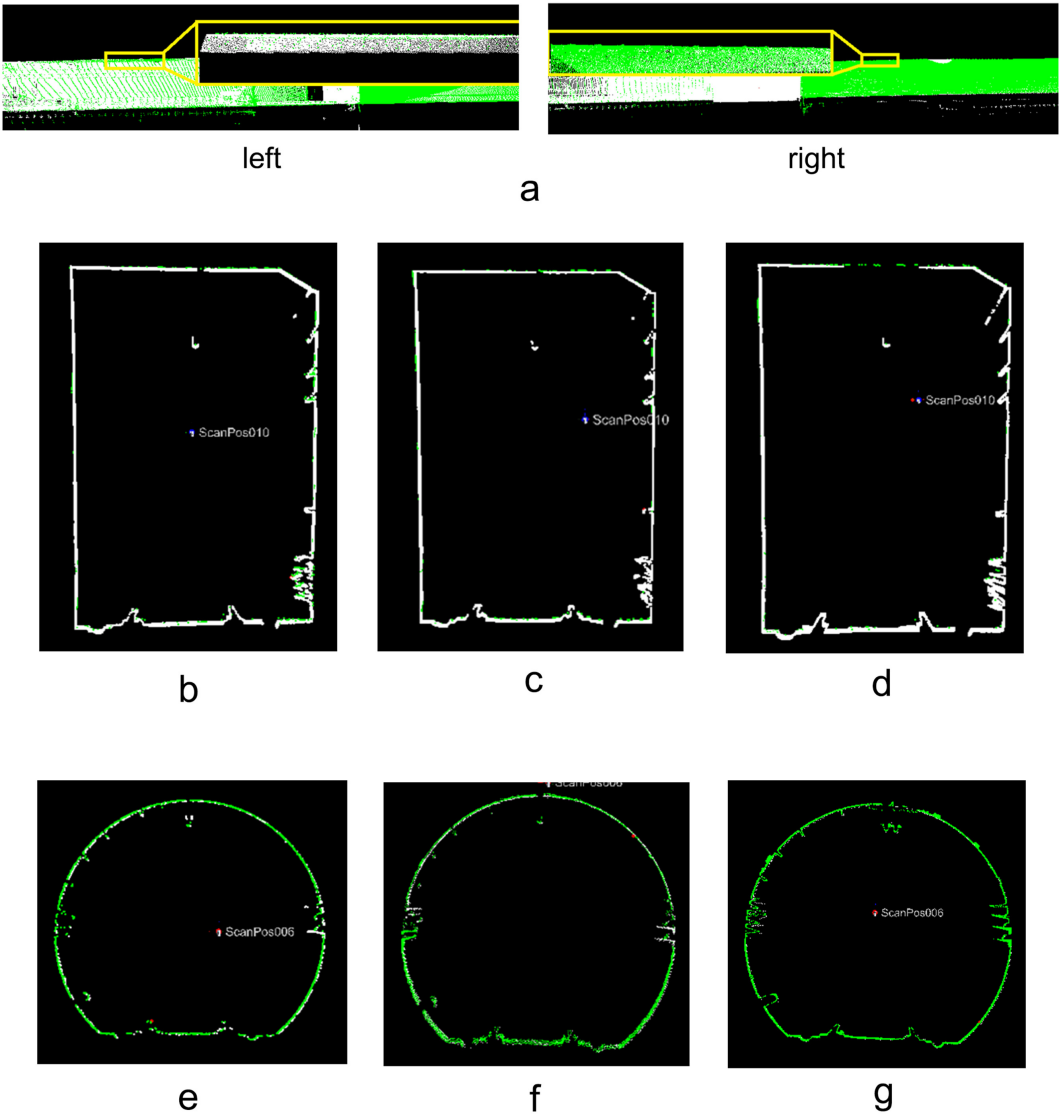
Global registration results (AEKF + Central-axis constraint). (**a**) Overview; (**b**) Scans 1 and 4; (**c**) Scans 2 and 5; (**d**) Scans 3 and 6; (**e**) Scans 7 and 10; (**f**) Scans 8 and 11; (**g**) Scans 9 and 12.

As presented in Section 3.2, the central axes were extracted by a global extraction strategy using segment-wise fitting, which produced many axis segments. Those segments were employed to construct an additional observation model according to Section 3.3 to control the error accumulation. Fig 16 shows that the deviations between the two cross sections are less than those shown in Fig 10. The average deviation computed from the results in Table 5 decreases to 0.024 m, which is only half of the mean value of 0.0463 m that is calculated from Table 2.

**Table 5:** Deviations between check point pairs (AEKF+Central-axis constraint).

	ID	Deviation/m	ID	Deviation/m	ID	Deviation/m	ID	Deviation/m
Scans 1 and 4	1	0.022	6	0.035	11	0.014	16	0.012
	2	0.024	7	0.031	12	0.014	17	0.008
	3	0.022	8	0.033	13	0.013	18	0.01
	4	0.029	9	0.043	14	0.021	19	0.009
	5	0.033	10	0.017	15	0.009	20	0.04
								0.0244
Scans 2 and 5	1	0.017	6	0.022	11	0.017	16	0.013
	2	0.02	7	0.021	12	0.01	17	0.024
	3	0.018	8	0.023	13	0.006	18	0.012
	4	0.025	9	0.019	14	0.01	19	0.017
	5	0.025	10	0.016	15	0.006	20	0.004
								0.0174
Scans 3 and 6	1	0.02	6	0.021	11	0.026	16	0.031
	2	0.041	7	0.02	12	0.027	17	0.035
	3	0.014	8	0.017	13	0.026	18	0.045
	4	0.017	9	0.02	14	0.028	19	0.018
	5	0.012	10	0.025	15	0.032	20	0.019
								0.0261
Scans 7 and 10	1	0.02	6	0.027	11	0.027	16	0.018
	2	0.032	7	0.022	12	0.008	17	0.026
	3	0.029	8	0.02	13	0.026	18	0.022
	4	0.028	9	0.025	14	0.018	19	0.031
	5	0.034	10	0.025	15	0.026	20	0.012
								0.0246
Scans 8 and 11	1	0.028	6	0.02	11	0.041	16	0.0320
	2	0.016	7	0.023	12	0.039	17	0.0370
	3	0.013	8	0.048	13	0.025	18	0.0230
	4	0.025	9	0.055	14	0.022	19	0.0340
	5	0.021	10	0.036	15	0.039	20	0.0140
								0.0316
Scans 9 and12	1	0.022	6	0.018	11	0.02	16	0.0300
	2	0.014	7	0.019	12	0.019	17	0.0250
	3	0.032	8	0.023	13	0.016	18	0.0130
	4	0.039	9	0.027	14	0.02	19	0.0220
	5	0.02	10	0.026	15	0.04	20	0.0170
								0.0242

## Conclusions

In this paper, we proposed a global registration approach that is based on an augmented extended Kalman filter and central-axis constraints. The point cloud registration was regarded as a stochastic system, so we utilized AEKF to produce accurate estimates of the rigid transformation parameters by eliminating the error accumulation caused by the pair-wise registration. Moreover, the central axis that was extracted from the scan was used to control the registration of multiple scans. The central axis of the subway tunnel was determined through a global extraction algorithm based on segment-wise quadratic curve fitting. We then derived the central-axis constraint as an additional observation model of AEKF to optimize the registration parameters between each pair of adjacent scans.

The proposed algorithm was implemented using a terrestrial laser scanning dataset that was acquired in a subway tunnel. The experimental results show that when multiple scans are aligned into a common coordinate frame, the consecutive implementation of pair-wise registration causes error accumulation (from 0.022 m to 0.046 m). The results also illustrate that the application of the global registration based on AEKF can reduce the error accumulation (from 0.046 m to 0.030 m). The proposed algorithm of global extraction based on segment-wise fitting was implemented on the experimental point clouds to impose the central-axis constraint and achieved an accuracy of 2 mm. The presented AEKF with the segment-wise axis constraint was shown to improve the accuracy of aligning multiple scans by 48% (0.024 m versus 0.046 m).

Because the extraction of the central axis is important to the global registration method, future work will focus on improving the robustness and applicability of our algorithm for the extraction of the tunnel’s central axis. Moreover, tunnels with more complex shapes and sharper curves will be considered in future work.
